# Cross‐sectional study of plasma phosphorylated tau 217 in persons without dementia

**DOI:** 10.1002/dad2.70107

**Published:** 2025-05-09

**Authors:** Toni T. Saari, Teemu Palviainen, Mikko Hiltunen, Sanna‐Kaisa Herukka, Tarja Kokkola, Sari Kärkkäinen, Mia Urjansson, Aino Aaltonen, Aarno Palotie, Heiko Runz, Jaakko Kaprio, Valtteri Julkunen, Eero Vuoksimaa, for FinnGen

**Affiliations:** ^1^ Institute for Molecular Medicine Finland (FIMM) Helsinki Institute of Life Science University of Helsinki Helsinki Finland; ^2^ Institute of Biomedicine University of Eastern Finland Kuopio Finland; ^3^ Institute of Clinical Medicine/Neurology University of Eastern Finland Kuopio Finland; ^4^ Department of Neurology Neurocenter Kuopio University Hospital Kuopio Finland; ^5^ Analytic and Translational Genetics Unit Department of Medicine Department of Neurology and Department of Psychiatry Massachusetts General Hospital Boston Massachusetts USA; ^6^ The Stanley Center for Psychiatric Research and Program in Medical and Population Genetics The Broad Institute of MIT and Harvard Cambridge Massachusetts USA; ^7^ European Molecular Biological Laboratories (EMBL) Heidelberg Germany

**Keywords:** Alzheimer's disease, apolipoprotein E, genome‐wide association study, plasma biomarkers, twins

## Abstract

**INTRODUCTION:**

Little is known about plasma phosphorylated tau 217 (p‐tau217) in individuals without a clinical diagnosis of Alzheimer's disease (AD). We studied associations of plasma p‐tau217 with age, sex, education, and genetic risk; estimated the heritability; and conducted a genome‐wide association study (GWAS).

**METHODS:**

A population‐based biobank recall study of 65‐ to 85‐year‐old twins (*N* = 697, mean [SD] age 76.2 [4.6] years; 53% women, 154 full pairs) excluding those with AD based on health registry data.

**RESULTS:**

Higher p‐tau217 level and likelihood of AD neuropathologic change (p‐tau217 > 0.42 pg/mL; evident in 39%) were associated with higher age and having an apolipoprotein E (*APOE*) ε4 allele. Heritability was 0.56 (95% confidence interval [CI]: 0.36–0.79) and GWAS indicated 45 single nucleotide polymorphisms (SNPs) (*p *< 5 × 10^−08^) centered around the *APOE* locus.

**DISCUSSION:**

Our results elucidate the characteristics and genetic associations of p‐tau217 in a population‐based setting. We found many 65‐ to 85‐year‐olds without a clinical diagnosis of AD to have AD neuropathologic change based on plasma p‐tau217.

**Highlights:**

- Plasma phosphorylated tau 217 (p‐tau217) is a promising biomarker of Alzheimer's disease (AD).
- We studied plasma p‐tau217 in a population‐based sample of 65‐ to ‐85‐year‐olds.
- We excluded those with a clinical diagnosis of AD.
- Older age and having an apolipoprotein E (*APOE*) ε4 allele were associated with higher plasma p‐tau217.
- Heritability of p‐tau217 was 56% and a genome‐wide association study (GWAS) implicated genes around the *APOE* region.

## 1 BACKGROUND

Advances in blood‐based plasma biomarkers of Alzheimer's disease (AD) hold a promise for cost‐effective early risk evaluation and diagnosis. Recently, plasma phosphorylated tau 217 (p‐tau217) has been reported to have accuracy comparable to cerebrospinal fluid (CSF) biomarkers for the determination of abnormal positron emission tomography (PET) amyloid.^1^ However, there is less information about plasma p‐tau217 in population‐based samples,^2^ and the role of genetic effects in accounting for interindividual differences of the plasma protein among persons without dementia is not known.

*Apolipoprotein E* (*APOE*) gene—with ε4 as a risk allele–and polygenic risk score of AD (ADPRS) based on genome‐wide association study (GWAS) explains only a fraction of the genetic variance of clinically defined AD.^3^ Twin studies can estimate the relative contribution of genetic and environmental effects by comparing the similarity between monozygotic (MZ, genetically identical at the sequence level) and dizygotic (DZ, genetically full siblings) twin pairs. Only one twin study has investigated the heritability of AD plasma biomarkers and found that about half of the variance in amyloid beta (Aβ)42 and Aβ40 was explained by additive genetic effects, whereas Aβ42/40 had zero heritability; also plasma total tau had 50% heritability.^4^ However, twin studies have not investigated the heritability of plasma p‐tau217 despite its promise as an accurate cost‐effective biomarker of both amyloid and tau positivity.^1^

The large case–control GWASs of AD have indicated over 80 loci.^5^ However, large samples have been reached by relaxing the criteria of clinical diagnosis and using proxy cases with a familial history of dementia, thus resulting in more general dementia findings than AD‐specific hits.^6^ There is a paradigm shift from studying the genetics of AD based on clinical symptom diagnostics to an increased use of the biological classification of amyloid and tau pathology, correspondingly requiring new GWAS studies.^7^ Compared to clinical or proxy cases, biomarkers are more objective and reproducible measures that capture the biological process of AD. They have the added benefit of improved statistical power using continuous measures with less measurement error compared to binary clinical diagnoses.^5^ GWASs of AD plasma biomarkers have identified *APOE*, beta‐secretase 1 (*BACE1*), presenilin 2 (*PSEN2*), and amyloid beta precursor protein (*APP*) for plasma Aβ ^8^ and microtubule associated protein tau (*MAPT*) gene for plasma total tau.^9^ Earlier GWASs on plasma p‐tau using p‐tau181 in fewer than 2000 individuals have implicated only the *APOE* locus,^10^, ^11^ and there are no reported GWAS data on p‐tau217.

Here, we conducted a population‐based twin study of plasma p‐tau217 to (1) study the general characteristics and distribution of p‐tau217, (2) estimate the relative importance of genetic and environmental effects, (3) investigate its associations with *APOE* and ADPRS, and (iv) conduct a GWAS of plasma p‐tau217.

## 2 METHODS

### 2.1 Participants and measures

We identified twins born in 1938–1957 and included in the biobank of the Finnish Institute for Health and Welfare (THL) from the population‐based older Finnish Twin Cohort (FTC) study.^12^ After excluding those individuals with AD, other neurodegenerative diseases or other cognition‐affecting diseases based on medical records from all hospitals in Finland, 2718 individuals (65‐ to 85 years old) were invited to participate in the TWINGEN study (see Supplement 1 for details).^13^ A total of 830 participants (31%) returned signed informed consents for participation in TWINGEN and they were contacted by phone to verify the suitability and availability for an in‐person visit to one of the six study sites across Finland. Finally, 697 individuals (84% of those who returned signed consents) had an in‐person study visit in March 2023 to November 2023, yielding a participation rate of 26% (697/2718). All participants were of European ancestry.

Education was based on self‐report by questionnaire. Cognitive status was based on validated telephone assessment of dementia (TELE) instrument.^14^ Genome‐wide genotyping was used to define *APOE* genotype,^15^ and to calculate ADPRS.^16^ (See supplementary material for details.) We chose the ADPRS balancing the proportion of variance explained, total sample size, and the proportion of clinically confirmed cases and controls.^6^ To date there is no AD PRS based on biologically defined cases.

After ethical approval, all participants provided written informed consent (see supplementary material for details). We followed Strengthening the Reporting of Observational Studies in Epidemiology (STROBE) and STrengthening the REporting of Genetic Association Studies (STREGA) guidelines.

### 2.2 Blood sample and biomarker determination

Non‐fasting blood samples were drawn at one of the six study sites mostly between 9:00 a.m. and 3:00 p.m. Plasma was collected to Vacutainer 10 mL K2EDTA tube and then centrifuged at 1500 *g* for 10 min. After centrifugation, plasma was apportioned into 0.5 mL aliquots and first stored at −20°C and then moved to −80°C. These aliquots were sent to the Biomarker Laboratory of the University of Eastern Finland for the biomarker analyses. We quantified p‐tau217 in January 2024 using ALZpath Simoa pTau‐217 v2 Assay Kit (Quanterix, Ref# 104371).^1^ Prior to analyses, EDTA plasma samples were thawed, mixed, and centrifuged (10,000 x *g*, 5 min, +20°C).

### 2.3 Genotyping

DNA was extracted from blood/saliva samples. Chip genotyping (genotype calling algorithms in parentheses) was performed using Illumina Human610‐Quad v1.0 B and Human670‐QuadCustom v1.0 A (Illuminus), Illumina HumanCoreExome 12 v1.0 A, 12 v1.1 A, 24 v1.0 A, 24 v1.1 A, 24 v1.2 A (GenCall), or Affymetrix FinnGen Axiom arrays (AxiomGT1). Genotype quality control was done in three batches.^17^ Prephasing was performed with Eagle v2.3^18^ and imputation with Minimac3 v2.0.1 using the University of Michigan Imputation Server.^19^ Genotypes were imputed to Haplotype Reference Consortium release 1.1 reference panel.^20^

### 2.4 Statistical analyses

All analyses were done with R statistical software (version 4.3.2) in March to April 2024. Linear mixed models (*lme4* package)^21^ were used to investigate the associations of age, sex, education, and *APOE* (ε4 carriers vs non‐carriers and sub‐groups in post hoc analyses) or ADPRS with p‐tau217. We used a logistic regression model (*survey* package)^22^ by including all these factors in predicting AD neuropathologic change (ADNPC) based on a previously published >0.42 pg/mL cutoff for Aβ positivity,^1^ and cutoffs based on three range approach (multinomial regression with *svyVGAM* package)^23^ and tau positivity (binary) as secondary outcomes.^1^ Models with ADPRS included 10 first principal components (PCs) of genetic ancestry. Family structure was adjusted for in all models. (See supplementary material for details.)

Twin data including MZ and DZ twin pairs can be used to estimate the relative contribution of additive genetic (A) and common (C) and unique (E) environmental effects by decomposing phenotypic variance into these components. The A effects present narrow sense heritability (*h* ^2^ = additive genetic variance/phenotypic variance), C effects are all non‐genetic effects that make twins within a pair similar, and the E effects denote environmental effects that make twins within a pair different including measurement error. We used *OpenMx* structural equation package (2.21.8) in R (supplementary material).^24^

The genome‐wide association analyses were performed using a mixed linear model using age and sex as covariates with a sparse genetic relationship matrix as the random effect of the model controlling for familial and more distant genetic relatedness. The association testing was performed by regressing out the covariates from the phenotype and using the adjusted phenotype for the analyses, which were performed using the genetic complex trait analysis (GCTA)^25^ fastGWA function.^26^ After analysis, we filtered out all variants with effect allele frequency <1%, Hardy‐Weinberg equilibrium (HWE) *p*‐value < 1e‐06, and imputation quality <0.7. The extended Simes test, GATES,^27^ was used to perform gene‐based analyses. Thresholds for statistical significance were *p* > 5 ^x^ 10^−8^ in the GWAS and *p* > 5 ^x^ 10^−6^ in the gene‐based tests. The regional plot was generated with LocusZoom.^28^ Finemapping for the genome‐wide significant loci was done using GCTA‐COJO^29^ right after the main analyses. The gene‐based heritability was calculated using a biological Knowledge‐based mining platform for Genomic and Genetic association Summary statistics using gEne Expression effective heritability estimator (KGGSEE‐EHE).^30^

**RESEARCH IN CONTEXT**

1. **Systematic review**: We used PubMed to search for studies of plasma phosphorylated tau 217 (p‐tau217) and found no genome‐wide association studies or family studies reporting its heritability. Plasma p‐tau217 has a high accuracy for capturing Alzheimer's disease (AD)–related pathologic change but population‐based studies in individuals without AD are needed.
2. **Interpretation**: The heritability estimate of plasma p‐tau217 was 56% and the GWAS implicated multiple SNPs. Higher age and having apolipoprotein E (*APOE*) ε4 allele were related to higher plasma p‐tau217. Our results from a population‐based sample support the use of plasma p‐tau217 in detecting AD neuropathologic change and its use in future studies of the molecular genetics of AD.
3. **Future directions**: Future studies should investigate the associations of plasma p‐tau217 with cognition and neurodegenerative diseases in real‐world data. Our genome‐wide association analysis encourages using plasma p‐tau217 in future studies investigating the molecular genetics of AD.

## 3 RESULTS

### 3.1 Descriptive statistics and associations of age, sex, and education with p‐tau217

**FIGURE 1:**
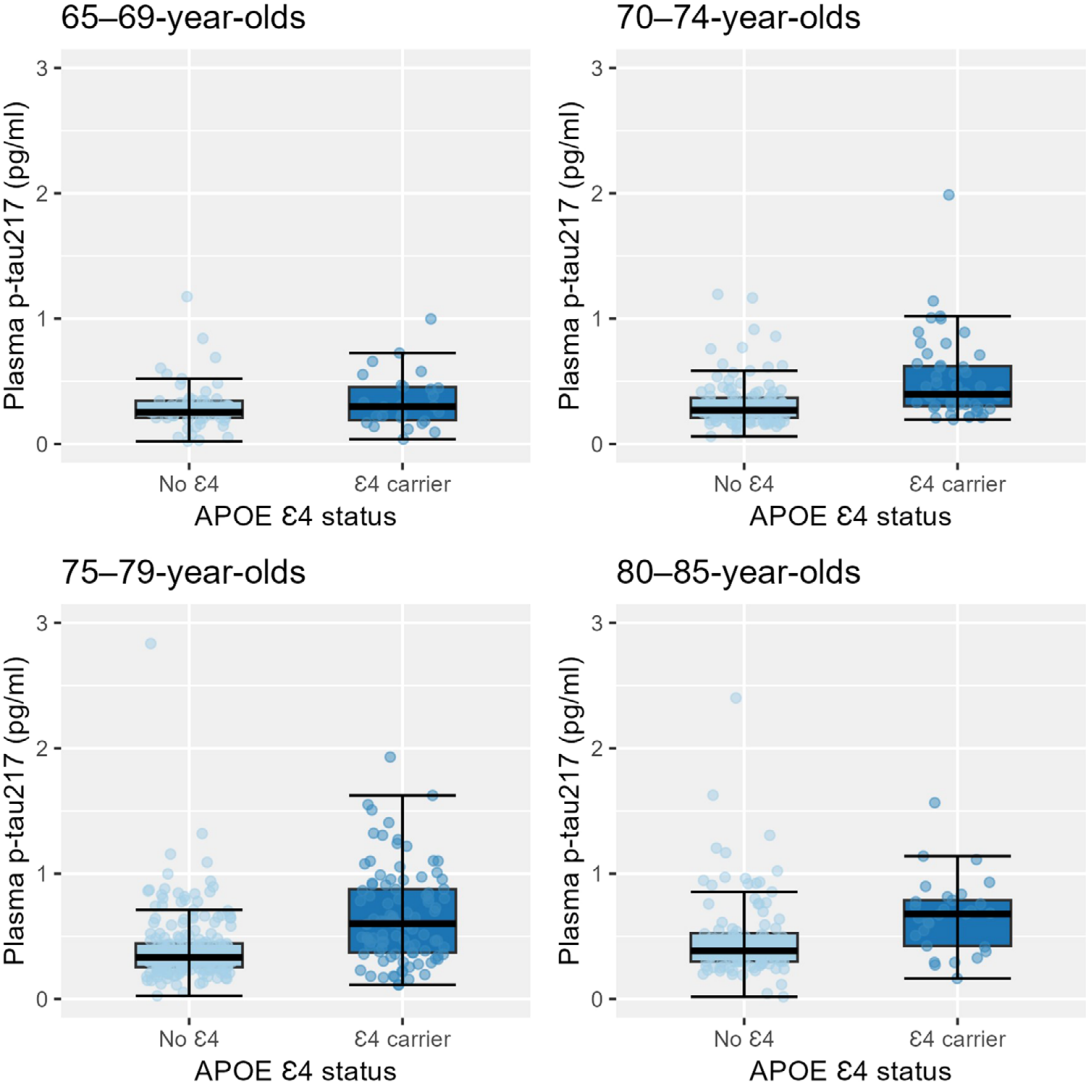
Phosphorylated tau 217 immunoassay levels in *APOE* ε4 carriers versus non‐carriers by age group (*n* = 695). *APOE*, apolipoprotein E.

Mean age (SD) of participants was 76.2 (4.6) years, 57% (*n* = 398) were women, 44% (*n* = 308) were from full twin pairs, and 389 were studied without their co‐twin. Values of p‐tau217 (*n* = 696) ranged from 0.02 to 2.84 pg/mL, the mean (SD) being 0.45 (0.31) pg/mL (Table 1, Figure S1). Age was positively associated with p‐tau217 level (*r* = 0.24, *p* < 0.001) (Figure 1, Table S1). Sex and education were not associated with p‐tau217 level (Table 1, Table S2).

**TABLE 1:** Sample demographics.

	All	Men	Women	MZ	DZ^c^	Uncertain zygosity
*N* (full twin pairs)	697 (154)	299 (55)	398 (99)	240 (81)	450 (73)	7 (0)
Age, mean (SD), years	76.17 (4.57)	76.65 (4.38)	75.81 (4.68)	75.24 (5.13)	76.63 (4.17)	78.47 (3.50)
Education, median (IQR), years	10 (7–13)	10 (7–18)	10 (7–13)	10 (7–13)	10 (7–13)	7 (7–10)
TELE, mean (SD), score	18.87 (1.17)	18.7 (1.22)	18.99 (1.12)	18.79 (1.13)	18.9 (1.2)	18.93 (0.45)
TELE <16^a^, no. (%)	27 (3.9)	13 (4.4)	14 (3.5)	11 (4.6)	16 (3.6)	0 (0)
*APOE* ε4 carriers, no. (%)	203 (29.2)	87 (29.2)	116 (29.1)	75 (31.4)	127 (28.2)	1 (14.3)
p‐tau217, mean (SD), pg/mL	0.45 (0.31)	0.47 (0.32)	0.43 (0.31)	0.43 (0.3)	0.46 (0.32)	0.42 (0.40)
p‐tau217 >0.42^b^, no. (%)	269 (38.6)	125 (41.9)	144 (36.2)	85 (35.6)	182 (40.4)	2 (28.6)
p‐tau217 <0.40^b^, no. (%)	407 (58.5)	162 (54.4)	245 (61.6)	144 (60.3)	258 (57.3)	5 (71.4)
p‐tau217 = 0.40–0.63,^b^ no. (%)	148 (21.3)	65 (21.8)	83 (20.9)	58 (24.3)	90 (20)	0 (0)
p‐tau217 >0.63^b^, no. (%)	141 (20.3)	71 (23.8)	70 (17.6)	37 (15.5)	102 (22.7)	2 (28.6)

Abbreviations: *APOE*, apolipoprotein E; DZ, dizygotic; IQR, interquartile range; MZ, monozygotic; p‐tau217, phosphorylated tau 217 immunoassay; SD, standard deviation; TELE, telephone assessment of dementia.

^a^ Cut‐point of cognitive impairment from Järvenpää et al. (2002).

^b^ Cut‐points from Ashton et al. (2024): >0.42 is a threshold for amyloid positivity, <0.40, 0.40–0.63, and >0.63 are thresholds for the three range approach for classifying individuals into low, intermediate, and high probability of amyloid positivity.

^c^ DZ twins included 313 (53 full pairs) from same‐sex twin pairs and 137 (20 full pairs) from opposite‐sex twin pairs. One participant had missing data in p‐tau217, one participant had missing data in *APOE*, and two participants had missing data in TELE.

### 3.2 ACE models for estimating the heritability and *APOE* and ADPRS associations with p‐tau217

Intra‐pair correlations were 0.55 (95% confidence interval [CI]: 0.37–0.69) in MZ and 0.24 (95% CI: 0.02–0.46) in DZ pairs. AE model with a heritability estimate of 0.56 (95% CI: 0.36–0.79) was the best‐fitting model (Table 2). *APOE* ε4 carriers had significantly higher p‐tau217 levels (mean [SD] = 0.58 [0.35] pg/mL) compared to non‐carriers (mean [SD] = 0.39 [0.27] pg/mL) (*b* = 0.41, 95% CI: 0.32–0.51, *p *< 0.001, *R* ^2 ^= 0.08) (Figure 1, Table S2). ADPRSs with *APOE* (*b* = 0.03, 95% CI: (−0.02)–0.08, *p* = 0.193, *R* ^2 ^= 0.001, Table S3) or without *APOE* (*b* = 0.02, 95% CI: [−0.02–0.07, *p* = 0.307, *R* ^2 ^= 0.0005, Table S4]) were not significantly related to p‐tau217 level.

**TABLE 2:** Model fitting results for estimating the relative proportion of genetic and environmental effects on phosphorylated tau 217.

Model	ep	−2LL	df	∆df	∆‐2LL	*p*	AIC	A (95% CI)	C (95% CI)	E (95% CI)
Saturated	10	796.18	284	NA	NA	NA	816.18	NA	NA	NA
ACE	4	801.98	290	6	5.8	0.446	809.98	0.69 (0.19–1.28)	−0.11 (−0.6–0.3)	0.43 (0.32–0.61)
**AE**	**3**	**802.25**	**291**	**1**	**0.27**	**0.599**	**808.25**	**0.56 (0.36–0.79)**	**NA**	**0.45 (0.33**–**0.61)**
CE	3	809.25	291	1	7.27	0.007	815.25	NA	0.39 (0.23–0.58)	0.61 (0.49–0.77)
E	2	833.33	292	2	31.35	< 0.001	837.33	NA	NA	1 (0.85–1.18)

*Note*: The estimate can be negative because we estimated A, C, and E components directly from the variances/covariances without any constraints. ACE model was compared against the saturated model. AE, CE, and E models were tested against the ACE model. Models included 77 full monozygotic and 70 full dizygotic twin pairs, and the best‐fitting model has been bolded.

Abbreviations: ∆‐2LL, change in minus two log likelihood; ∆df, change in degrees of freedom; –2LL, minus two log likelihood; A, additive genetic effects; AIC, Akaike's information criterion; C, common environmental effects; CI, confidence interval; df, degrees of freedom; E, unique environmental effects; ep, number of estimated parameters; NA, not applicable.

### 3.3 Prevalence and predictors of ADNPC based on abnormal p‐tau217

We used categorical p‐tau217 to indicate ADNPC based on previously published cutoffs for Aβ and tau positivity.^1^ The prevalence of ADNPC was 38.6% using >0.42 pg/mL cutoff indicative of Aβ positivity and was higher as a function of older age from 20.7% in 65‐ to 69 year olds to 50.7% in 80‐ to 85 year old (Table 1, Table S1). Older age (odds ratio [OR] = 1.15, 95% CI: 1.10–1.20, *p *< 0.001) and having *APOE* ε4 allele (OR = 4.53, 95% CI: 3.10–6.62) were predictors of amyloid positivity (Table S5). ADPRSs with (OR = 1.12. 95% CI: 0.95–1.33, Table S6) or without *APOE* (OR = 1.09, 95% CI: 0.91–1.30, Table S5) were not related to greater odds of amyloid positivity.

The association of *APOE* was even greater when using a three range approach (OR = 7.22, 95% CI: 4.50–11.59, *p *< 0.001, Table S7) contrasting those with low (p‐tau217 <0.40 pg/mL) and high (p‐tau217 >0.63 pg/mL) probability of amyloid positivity, and when using >0.64 pg/mL binary cutoff indicative of tau positivity (OR = 5.13, 95% CI: 3.32–7.91, *p *< 0.001, Table S8). The prevalence of tau positivity was 19.6% when using >0.64 pg/mL cutoff (Table S1). Sex and education were not associated with abnormal levels of p‐tau217 with any cutoffs (Tables S5–S8).

### 3.4 Genome‐wide association analysis of p‐tau217 plasma protein levels

**FIGURE 2:**
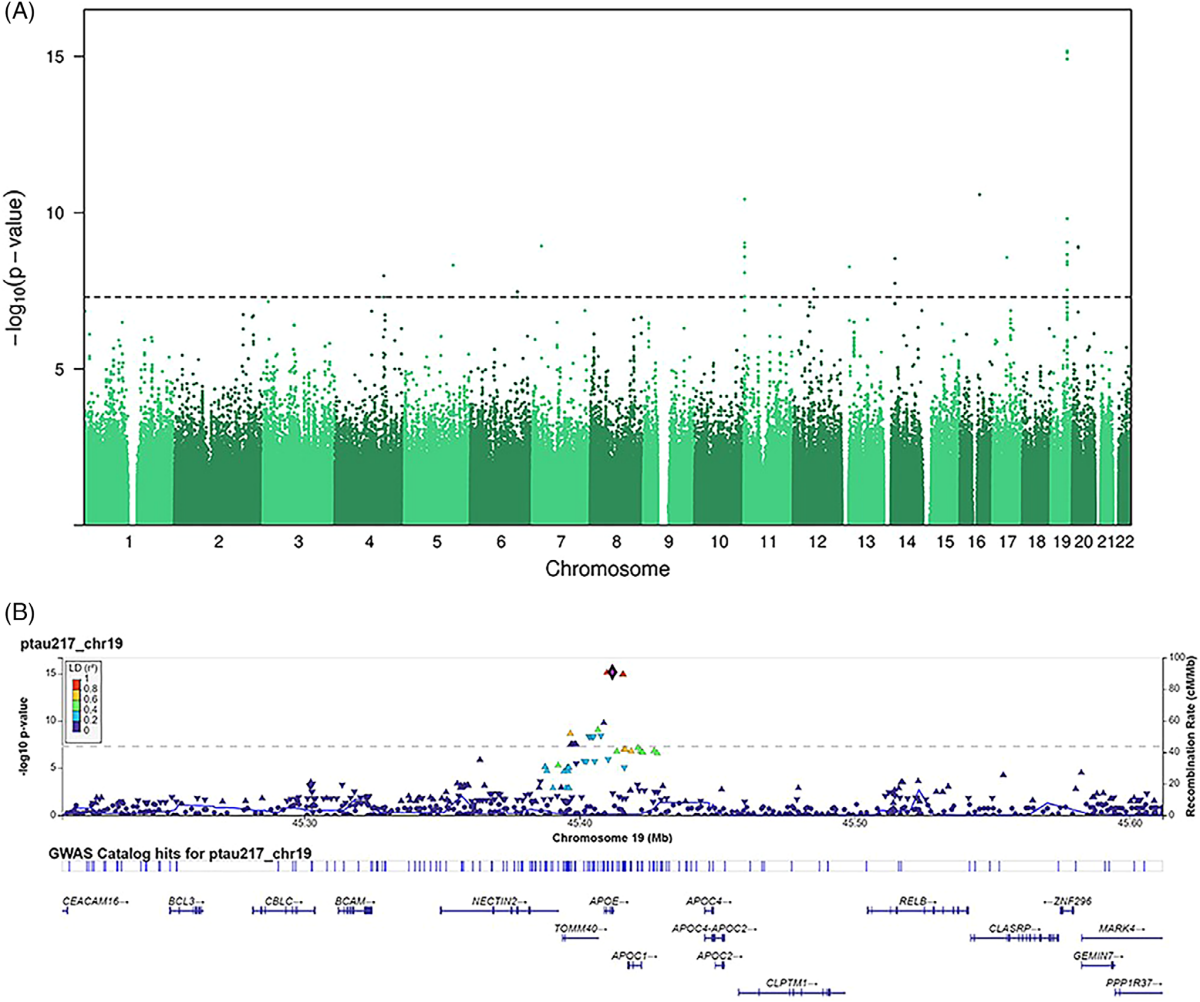
Genome‐wide association analysis of p‐tau217. (A) Manhattan plot for the genome‐wide association analysis of p‐tau217. (B) Regional plot in the *APOE* locus in Chromosome 19 for the SNP associations with p‐tau217 with rs429358 as a reference. *APOE*, apolipoprotein E; p‐tau217, phosphorylated tau 217; SNP, single nucleotide polymorphism.

Genomic inflation factor *λ* was 1.01, indicating no inflation in *p*‐values caused by population stratification or sample relatedness (Figure S2). We found a total of 45 lead variants with genome‐wide significant (*p* > 5 ^x^ 10^−8^) associations with p‐tau217 blood levels (Figure 2A, Table 3, Table S9); more than a third (17/45) were in chromosome 19 in the *APOE* region (Figure 2B). The top hit was rs429358, a single nucleotide polymorphism (SNP) that is used to define *APOE* ε4 carrier status (presence of C allele, *b* = 0.19, SE = 0.02, *p *= 5 ^x^ 10^−16^). In contrast, rs7412 (T allele indicating the presence of the AD protective ε2 allele, *b* = −0.10, SE = 0.04, *p *= 0.005) was not among the top SNPs. Fine mapping results indicated that associated genes in chromosome 19 are linked with each other, suggesting a single SNP, rs429358 (*b* = 0.19, SE = 0.02, *p* = 1 ^x^ 10^−14^), being putatively causal for p‐tau levels. In addition, in chromosomes 6 and 11, independent putatively causal SNPs were rs9402117 (*b* = 0.28, SE = 0.05, *p* = 6 ^x^ 10^−8^), and rs115553322 (*b* = 0.44, SE = 0.07, *p* = 1 ^x^ 10^−10^), respectively.

**TABLE 3:** SNPs with *p* < 5 ^x^ 10^−08^ from the genome‐wide association analysis of p‐tau217 (*n* = 695).

CHR	BP	SNP	A1	A2	*β* (95% CI)	SE	*p*	EAF	HWE *p*‐value	Info‐score
19	45411941	rs429358	C	T	0.19 (0.14–0.24)	0.02	6.790 × 10^−16^	0.15	0.431	0.996
19	45410002	rs769449	A	G	0.19 (0.15–0.24)	0.02	7.525 × 10^−16^	0.15	0.386	0.998
19	45415713	rs10414043	A	G	0.19 (0.14–0.24)	0.02	1.233 × 10^−15^	0.15	0.353	0.993
19	45415935	rs7256200	T	G	0.19 (0.14–0.24)	0.02	1.233 × 10^−15^	0.15	0.353	0.993
16	54004229	rs116290784	T	C	0.52 (0.37–0.68)	0.08	2.627 × 10^−11^	0.01	0.820	0.882
11	1653718	rs115553322	A	G	0.44 (0.31–0.57)	0.07	3.731 × 10^−11^	0.01	0.696	0.915
19	45408836	rs405509	T	G	0.11 (0.08–0.15)	0.02	1.565 × 10^−10^	0.43	0.390	0.996
19	45406673	rs10119	A	G	0.12 (0.08–0.16)	0.02	8.966 × 10^−10^	0.26	0.321	0.985
11	1650080	rs140772159	A	G	0.38 (0.26–0.51)	0.06	9.320 × 10^−10^	0.02	0.580	0.933
7	24971877	rs147827528	C	T	0.51 (0.35–0.67)	0.08	1.177 × 10^−09^	0.01	0.516	0.893
20	16447628	rs183298906	A	G	0.49 (0.33–0.65)	0.08	1.231 × 10^−09^	0.01	0.317	0.926
11	1493389	rs749878214	T	C	0.38 (0.26–0.51)	0.06	1.255 × 10^−09^	0.02	0.716	0.988
11	1536741	rs146099176	A	G	0.38 (0.26–0.51)	0.06	1.255 × 10^−09^	0.02	0.704	0.900
20	16595515	rs150906525	G	A	0.45 (0.30–0.59)	0.07	1.330 × 10^−09^	0.01	0.317	0.904
19	45396665	rs59007384	T	G	0.12 (0.08–0.16)	0.02	2.209 × 10^−09^	0.21	0.506	0.994
11	1614455	rs143478050	G	A	0.30 (0.20–0.40)	0.05	2.556 × 10^−09^	0.03	0.649	0.989
17	38840826	rs117627297	A	G	0.42 (0.28–0.56)	0.07	2.672 × 10^−09^	0.01	0.457	0.932
14	31181415	rs75349909	C	T	0.38 (0.25–0.50)	0.06	2.927 × 10^−09^	0.02	0.369	0.896
19	45407788	rs7259620	A	G	−0.10 (−0.14–0.07)	0.02	3.741 × 10^−09^	0.53	0.425	0.994
19	45403412	rs1160985	T	C	−0.10 (−0.14—0.07)	0.02	4.602 × 10^−09^	0.53	0.467	0.996
19	45403858	rs760136	G	A	−0.10 (−0.14—0.07)	0.02	4.602 × 10^−09^	0.53	0.471	0.996
19	45404431	rs741780	C	T	−0.10 (−0.14—0.07)	0.02	4.602 × 10^−09^	0.53	0.471	0.996
19	45404972	rs1038025	C	T	−0.10 (−0.14—0.07)	0.02	4.602 × 10^−09^	0.53	0.471	0.996
19	45405062	rs1038026	G	A	−0.10 (−0.14—0.07)	0.02	4.602 × 10^−09^	0.53	0.471	0.995
5	133599786	rs11742455	T	C	0.42 (0.28–0.56)	0.07	4.833 × 10^−09^	0.01	0.661	0.920
13	20936498	rs17080846	T	C	0.42 (0.28–0.56)	0.07	5.309 × 10^−09^	0.01	0.317	0.967
11	1491298	rs554200731	T	C	0.36 (0.23–0.48)	0.06	8.231 × 10^−09^	0.02	0.704	0.963
4	134053110	rs115976772	A	G	0.45 (0.29–0.60)	0.08	1.056 × 10^−08^	0.01	0.439	0.845
14	31110189	rs77863412	A	G	0.28 (0.18–0.38)	0.05	1.838 × 10^−08^	0.03	0.350	0.904
12	56724591	rs147236029	G	T	0.30 (0.20–0.41)	0.05	2.758 × 10^−08^	0.02	0.403	0.938
19	45396899	rs157584	T	C	0.10 (0.06–0.13)	0.02	2.970 × 10^−08^	0.46	0.585	0.995
19	45397512	rs157585	A	C	0.10 (0.06–0.13)	0.02	2.970 × 10^−08^	0.46	0.626	0.995
19	45398264	rs157588	C	T	0.10 (0.06–0.13)	0.02	2.970 × 10^−08^	0.46	0.622	0.995
19	45398716	rs157590	A	C	0.10 (0.06–0.13)	0.02	2.970 × 10^−08^	0.46	0.599	0.993
6	129633534	rs9402117	C	T	0.28 (0.18–0.37)	0.05	3.389 × 10^−08^	0.02	0.468	0.992
6	129635800	rs2306942	A	G	0.28 (0.18–0.37)	0.05	3.389 × 10^−08^	0.02	0.470	0.992
6	129638768	rs9321160	A	G	0.28 (0.18–0.37)	0.05	3.389 × 10^−08^	0.02	0.470	0.992
6	129640374	rs3798660	C	G	0.28 (0.18–0.37)	0.05	3.389 × 10^−08^	0.02	0.470	0.992
6	129667406	rs9372925	A	T	0.28 (0.18–0.37)	0.05	3.389 × 10^−08^	0.02	0.389	0.998
6	129673689	rs9385488	C	T	0.28 (0.18–0.37)	0.05	3.389 × 10^−08^	0.02	0.388	0.999
6	129674757	rs3813367	T	G	0.28 (0.18–0.37)	0.05	3.389 × 10^−08^	0.02	0.388	0.998
6	129700998	rs17057200	T	C	0.28 (0.18–0.37)	0.05	3.389 × 10^−08^	0.02	0.431	0.988
11	1457099	rs568790642	G	C	0.33 (0.21–0.44)	0.06	4.803 × 10^−08^	0.02	0.585	0.959
4	134009465	rs79072289	G	A	0.41 (0.27–0.56)	0.08	4.973 × 10^−08^	0.01	0.437	0.836
6	129628090	rs73585572	T	G	0.28 (0.18–0.38)	0.05	4.982 × 10^−08^	0.02	0.522	0.991

*Note*: Genome positions are in build 37 (hg19/GRCh37).

Abbreviations: A1, effect allele; A2, non‐effect allele; BP, base‐pair position; CHR, chromosome; EAF, effect allele frequency; HWE, Hardy–Weinberg equilibrium; SE, standard error; SNP, single nucleotide polymorphism.

Gene‐based tests indicated five genes (*p* > 5 ^x^ 10^−6^) in the *APOE* region including *APOE*, translocase of outer mitochondrial membrane 40 *(TOMM40)*, apolipoprotein C1 *(APOC1)*, nectin cell adhesion molecule 2 *(NECTIN2)*, and apolipoprotein C1 pseudogene 1 *(APOC1P1)* (Table S10). For the chromosome 19 loci, gene‐based heritabilities were following: *APOE* = 9.24% (SE 2.31%), *TOMM40* = 9.24% (SE 2.31%), *APOC1* = 9.07% (SE 2.29%), *NECTIN2* = 5.00% (SE 1.71%), and *APOC1P1* = 3.82% (SE 1.50%). GCTA‐based heritability estimate of p‐tau217 was 49.2% (SE 43%).

### 3.5 Post hoc analyses on *APOE* genotype subgroups

We investigated the associations of *APOE* risk allele ε4 (ε3/ε4 and ε4/ε4 groups separately), protective ε2 allele (combining four individuals with ε2/ε2 and 52 individuals with ε2/ε3), and the combination of risk and protective alleles ε2/ε4 on p‐tau217 (ε3/ε3 as a reference group and age, sex, and education as covariates). Participants with one (*n* = 174, *b* = 0.40, 95% CI: 0.30–0.50, *p *< 0.001) or two (*n* = 12, *b* = 0.80, 95% CI: 0.49–1.15, *p *< 0.001) ε4 alleles had a significantly higher p‐tau217 level than participants with two ε3 alleles (*n* = 436). Having the ε2 allele was not associated with p‐tau217 (*n* = 56, *b* = −0.11, 95% CI: −0.27–0.05, *p *= 0.182). Finally, the ε2/ε4 group did not differ (*n* = 17, *b* = 0.11, 95%CI: −0.17–0.40, *p *= 0.435) from the ε3/ε3 group.

## 4 DISCUSSION

We found in a population‐based sample of 65‐ to 85 year olds that older age was related to higher plasma p‐tau217 levels. We also found that older age was associated with a higher likelihood of having abnormal levels of p‐tau217, indicative of AD neuropathologic change. Plasma p‐tau217 has been recognized as an early changing core biomarker—indicative of both plaques and tangles and reflecting AD neuropathologic change—in the latest criteria for diagnosis and staging of AD by the Alzheimer's Association workgroup.^31^, ^32^

We also found that a substantial proportion of the variance in p‐tau217 was explained by additive genetic effects with a heritability estimate of 56%. To our knowledge, only one twin study—not including p‐tau217—has investigated the heritability of blood‐based AD biomarkers.^4^ We found that age and *APOE* genotype, but not ADPRS (based on cases having clinical diagnosis of AD), were significant predictors of continuous p‐tau217 levels but also ADNPC, as indicated by abnormal levels of plasma p‐tau217 using published cutoffs for amyloid and tau positivity.^1^ For the ADPRS, we chose to use the score based on Lambert et al.^16^ The larger sample sizes in later GWASs of AD—all with smaller *r*‐squared than the 0.09 in Lambert et al.—have included also proxy cases where AD status is not based on clinical diagnosis but on family history, thus PRSs from these studies reflect a more general dementia phenotype that is not specific to AD.^6^

To our knowledge, this was the first GWAS of plasma p‐tau217 where we found a total of 45 significant SNPs. These implicated the *APOE* gene but also other chromosome 19 genes in the same region: *TOMM40, APOC1, NECTIN2, and APOC1P1*. These genes have been linked previously with AD and dementia phenotypes. Except for *APC1P1*, these genes are protein coding, with *APOE* and *APOC1* involved in lipid/cholesterol metabolism, *TOMM40* involved in protein transport into mitochondria, and *NECTIN2* as entry for herpesvirus and involved in cell‐to‐cell spreading of viruses. *APOE*, *APOC1*, and *TOMM40* are in high linkage disequilibrium, but there is evidence that having additional risk alleles in *APOC1* and *TOMM40* increases the risk of AD over and above the risk of the *APOE* ε4 allele.^33^ Together, *APOE*, *APOC1*, and *NECTIN2* haplotypes confirm a risk of AD over and above *APOE*.^34^ In chromosome 19, our results suggested only rs429358 as a putative causal SNP, indicating the importance of the *APOE* gene for plasma p‐tau217 levels. In addition, we found two putative causal SNPs, rs9402117 and rs115553322, in chromosomes 6 and 11, respectively, but these have not been indicated earlier to be associated with AD or dementia, and no genes in these chromosomes were implicated in the gene‐based analyses.

The association of the *APOE* gene with AD and its cognitive and biomarkers is well established, but the largest GWASs using case–control designs, including proxy cases, have not implicated other *APOE* region genes that were associated with p‐tau217 in our study.^35^, ^36^, ^37^ However, a case–control GWAS (with family history of dementia as exclusion criteria in cases) with 9 of 13 significant SNPs in the *APOE* region implicated *APOE*, *APOC1*, *TOMM40*, and *NECTIN2* in the prediction of clinical diagnosis of AD in the Chinese population.^38^ GWAS of p‐tau determined from CSF samples indicated four loci including *APOE*.^39^ The *APOE* ε4 allele determining rs429358‐C was associated with higher CSF p‐tau levels, whereas rs7412‐T was associated with lower p‐tau.^39^ In our study, rs429358‐C was associated with higher p‐tau217 but rs7412‐T was not related to p‐tau217 levels.

Earlier GWASs of plasma p‐tau181 have indicated no significant SNPs other than in the *APOE* gene.^10^, ^11^ In line with our results, GWASs on plasma ApoE found significant SNPs in the same *APOE* region genes as our study, and gene‐based tests indicated that *APOE*, *APOC1*, *TOMM40*, and *NECTIN2* were related to plasma ApoE concentration and risk of AD.^40^ GWAS of possible preclinical neurodegenerative disease blood biomarkers of caspase‐3‐cleaved (TAU‐C) and ADAM‐10 cleaved tau (TAU‐A) found only one significant SNP for each of these biomarkers: *APOC1* (rs10414043) for TAU‐A and *APOE* (rs429358) for TAU‐C.^41^ These SNPs were our third and first top SNPs in relation to p‐tau217, respectively.

In the biological AT(N) classification, biomarker classification and cognitive staging are independent and AD is defined simply on the biomarker status of amyloid and tau positivity.^7^ Prevalence of 39% for amyloid positivity (p‐tau217 >0.42 pg/mL) in our study was very similar to the prevalence of PET amyloid positivity in a population‐based sample of cognitively healthy persons,^42^ and the age‐specific prevalence figures were similar to a meta‐analysis^43^ and a pooled study of 85 cohorts^44^ for amyloid positivity from CSF or PET in individuals without dementia. Using the biological definition of A+T+, the prevalence of ADNPC in our sample was estimated to be ∼20% based on the three‐range and tau positivity cutoffs. Given our sample excluding individuals with a clinical diagnosis of AD, the substantial proportion of amyloid‐ and tau‐positive individuals indicates that plasma p‐tau217 is a useful biomarker for determining AD biological continuum independent of cognitive staging and can guide in screening, diagnosis, and prognosis of AD. Together with cognitive staging, p‐tau217 may help in detecting preclinical (biomarker positive and normal cognition) and prodromal (biomarker positive and mild cognitive impairment) AD.

### 4.1 Limitations and strengths

Despite our exclusion based on health care registries, about 4% of our sample had cognitive impairment according to telephone screening. Individuals with cognitive impairment may have undiagnosed clinical AD despite not having the diagnosis yet, but we note that scores below the cutoff for cognitive impairment in this telephone‐administered instrument may also arise for reasons other than dementia (e.g., hearing problems or environmental disturbances during the interview).

A limitation of our study was that we did not adjust for conditions that could affect plasma biomarker levels, such as reduced kidney function, obesity, and cardiovascular conditions.^45^ Recent studies have shown elevated levels of plasma p‐tau217 in individuals with worse kidney function,^2^, ^46^, ^47^ but other studies have found no significant associations between kidney function and plasma p‐tau217.^48^, ^49^ The discrepant results may relate to differences in study samples and measurement. Moreover, although the areas under the receiver‐operating characteristic (ROC) curve have been ≥0.90 for plasma p‐tau217 in detecting CSF or PET amyloid positivity,^1^, ^50^ the cutoff used in this study requires further validation. In addition, other cutoffs for ALZpath p‐tau217 have been published recently. Our study did not include CSF or PET measures of Aβ, which could be used to validate the p‐tau217 cutoff in population‐based settings, but the focus of our study was in studying the general characteristics of p‐tau217, its heritability, and genome‐wide associations rather than validation of the cutoffs.

The participation rate of our study was 26%, which can be considered adequate for a clinical biobank recall study requiring participants to travel to the study site for an in‐person visit. Strengths included a population‐based sample with the expected ≈30% prevalence of *APOE* ε4 carriership^15^ and health care register–based exclusion of those diagnosed with clinical AD and many other medical conditions affecting the central nervous system. We chose the ADPRS balancing the sample size, proportions of variance explained, and criteria of clinically confirmed cases and controls.^6^ To date there is no ADPRS based on biologically defined cases. We did not detect significant associations of ADPRS and *APOE* ε2 allele with p‐tau217 (although the direction of association was as expected), which is likely due to a relatively small sample size. Considering our GWAS, despite multiple significant SNPs associated with p‐tau217, our sample was relatively small even for a biomarker study, and we did not have access to a replication sample. However, our results encourage the use of plasma p‐tau217 in future GWAS as it is an easily scalable measure that captures the biology of AD.

## 5 CONCLUSION

This cross‐sectional population‐based study indicated that over half of the variance in p‐tau217 was explained by additive genetic effects in individuals without dementia. Age and *APOE*, but not sex, was associated with p‐tau217 and abnormal levels indicative of ADNPC. Based on previously published cutoffs, many—especially those who were 75‐ to 85 years old—had a high probability of ADNPC even though they had no diagnosis of AD. Our GWAS was able to detect polygenic signal for plasma p‐tau217 and findings implicated genes in the *APOE* region. Overall, our results elucidate the characteristics and genetic associations of p‐tau217 in a population‐based setting.

## CONFLICT OF INTEREST STATEMENT

A.P. is the Chief Scientific Officer of the FinnGen project, which is funded by 14 pharmaceutical companies. The FinnGen partner pharma companies are: AbbVie Inc., AstraZeneca UK Ltd, Biogen MA Inc., Bristol Myers Squibb (and Celgene Corporation & Celgene International II Sàrl), Genentech Inc., Merck Sharp & Dohme LCC, Pfizer Inc., GlaxoSmithKline Intellectual Property Development Ltd., Sanofi US Services Inc., Maze Therapeutics Inc., Janssen Biotech Inc, Novartis Pharma AG, Boehringer Ingelheim International GmbH, and Bayer. H.R. is a current employee of Insitro Inc., a former employee of Biogen, reports speaker's fees and travel payments from Roche and holds stock at Merck & Co and Biogen Inc. A.A. declares personal funding from the Finnish Cultural Foundation and Tauno Virtanen fund. E.V. reports Sigrid Jusélius Foundation and Research Council of Finland funding paid to the University of Helsinki. The authors declare no other competing financial or non‐financial interests. Author disclosures are available in the Supporting Information.

## CONSENT STATEMENT

Ethical approvals for the TWINGEN protocol were obtained from the Coordinating Ethics Committee of the Hospital District of Helsinki and Uusimaa (HUS) (number 16831/2022), and THL Biobank approved the research plan with the permission no: THLBB2022_83. All participants gave written informed consent before their participation and had the option to withdraw from the study at any point. One consent was given to the University of Helsinki twin study group and the other consent was for the THL biobank to allow data transfer from University of Helsinki to the THL biobank based on a data transfer agreement between the institutions.

## Supporting information



Supporting Information

Supporting Information

FinnGen author list

## Data Availability

TWINGEN data are stored at the THL Biobank for those participants who gave consent for transferring their data to the biobank. Data are available to qualified applicants from academia and companies; for details see https://thl.fi/en/research‐and‐development/thl‐biobank/for‐researchers/application‐process.
