# Supplementary material for: Cross‐sectional study of plasma phosphorylated tau 217 in persons without dementia
Source: Alzheimers Dement (Amst). 2025 May 9;17(2):e70107. doi: 10.1002/dad2.70107 (PMC12064337; doi:10.1002/dad2.70107)
Supplement: Supplementary file 3 — FinnGen author list [file DAD2-17-e70107-s002.docx]

FinnGen author list

| **FinnGen** |  |  |  |  |  |
| --- | --- | --- | --- | --- | --- |
| **Full Name** | **Affiliation** | **E-mail** | **Role 1** | **Role 2** |  |
| Aarno Palotie | Institute for Molecular Medicine Finland (FIMM), HiLIFE, University of Helsinki, Helsinki, Finland; Broad Institute of MIT and Harvard; Massachusetts General Hospital | aarno.palotie@helsinki.fi | **Steering Committee** | **Steering Committee** | |
| Mark Daly | Institute for Molecular Medicine Finland (FIMM), HiLIFE, University of Helsinki, Helsinki, Finland; Broad Institute of MIT and Harvard; Massachusetts General Hospital | mark.daly@helsinki.fi | **Steering Committee** | **Steering Committee** | |
| Bridget Riley-Gills | Abbvie, Chicago, IL, United States | bridget.rileygillis@abbvie.com | **Steering Committee** | **Pharmaceutical companies** | |
| Howard Jacob | Abbvie, Chicago, IL, United States | howard.jacob@abbvie.com | **Steering Committee** | **Pharmaceutical companies** | |
| Coralie Viollet | Astra Zeneca, Cambridge, United Kingdom | coralie.viollet@astrazeneca.com | **Steering Committee** | **Pharmaceutical companies** | |
| Slavé Petrovski | Astra Zeneca, Cambridge, United Kingdom | slav.petrovski@astrazeneca.com | **Steering Committee** | **Pharmaceutical companies** | |
| Chia-Yen Chen | Biogen, Cambridge, MA, United States | chiayen.chen@biogen.com | **Steering Committee** | **Pharmaceutical companies** | |
| Sally John | Biogen, Cambridge, MA, United States | sally.john@biogen.com | **Steering Committee** | **Pharmaceutical companies** | |
| George Okafo | Boehringer Ingelheim, Ingelheim am Rhein, Germany | george.okafo@boehringer-ingelheim.com | **Steering Committee** | **Pharmaceutical companies** | |
| Robert Plenge | Bristol Myers Squibb, New York, NY, United States | robert.plenge@bms.com | **Steering Committee** | **Pharmaceutical companies** | |
| Joseph Maranville | Bristol Myers Squibb, New York, NY, United States | joseph.maranville@bms.com | **Steering Committee** | **Pharmaceutical companies** | |
| Mark McCarthy | Genentech, San Francisco, CA, United States | mccarthy.mark@gene.com | **Steering Committee** | **Pharmaceutical companies** | |
| Rion Pendergrass | Genentech, San Francisco, CA, United States | penders2@gene.com | **Steering Committee** | **Pharmaceutical companies** | |
| Jonathan Davitte | GlaxoSmithKline, Collegeville, PA, United States | jonathan.m.davitte@gsk.com | **Steering Committee** | **Pharmaceutical companies** | |
| Kirsi Auro | GlaxoSmithKline, Espoo, Finland | kirsi.m.auro@gsk.com | **Steering Committee** | **Pharmaceutical companies** | |
| Simonne Longerich | Merck, Kenilworth, NJ, United States | simonne.longerich@merck.com | **Steering Committee** | **Pharmaceutical companies** | |
| Anders Mälarstig | Pfizer, New York, NY, United States | anders.malarstig@pfizer.com | **Steering Committee** | **Pharmaceutical companies** | |
| Anna Vlahiotis | Pfizer, New York, NY, United States | anna.vlahiotis@pfizer.com | **Steering Committee** | **Pharmaceutical companies** | |
| Katherine Klinger | Translational Sciences, Sanofi R&D, Framingham, MA, USA | katherine.klinger@sanofi.com | **Steering Committee** | **Pharmaceutical companies** | |
| Clement Chatelain | Translational Sciences, Sanofi R&D, Framingham, MA, USA | clement.chatelain@sanofi.com | **Steering Committee** | **Pharmaceutical companies** | |
| Matthias Gossel | Translational Sciences, Sanofi R&D, Framingham, MA, USA | matthias.gossel@sanofi.com | **Steering Committee** | **Pharmaceutical companies** | |
| Karol Estrada | Maze Therapeutics, San Francisco, CA, United States | kestrada@mazetx.com | **Steering Committee** | **Pharmaceutical companies** | |
| Robert Graham | Maze Therapeutics, San Francisco, CA, United States | rgraham@mazetx.com | **Steering Committee** | **Pharmaceutical companies** | |
| Dawn Waterworth | Janssen Research & Development, LLC, Spring House, PA, United States | dwaterwo@its.jnj.com | **Steering Committee** | **Pharmaceutical companies** | |
| Chris O´Donnell | Novartis Institutes for BioMedical Research, Cambridge, MA, United States | chris.odonnell@novartis.com | **Steering Committee** | **Pharmaceutical companies** | |
| Nicole Renaud | Novartis Institutes for BioMedical Research, Cambridge, MA, United States | nicole.renaud@novartis.com | **Steering Committee** | **Pharmaceutical companies** | |
| Tomi P. Mäkelä | HiLIFE, University of Helsinki, Finland, Finland | tomi.makela@helsinki.fi | **Steering Committee** | **University of Helsinki & Biobanks** | |
| Jaakko Kaprio | Institute for Molecular Medicine Finland (FIMM), HiLIFE, University of Helsinki, Helsinki, Finland | jaakko.kaprio@helsinki.fi | **Steering Committee** | **University of Helsinki & Biobanks** | |
| Minna Ruddock | Arctic biobank / University of Oulu | minna.ruddock@oulu.fi | **Steering Committee** | **University of Helsinki & Biobanks** | |
| Petri Virolainen | Auria Biobank / University of Turku / Hospital District of Southwest Finland, Turku, Finland | petri.virolainen@tyks.fi | **Steering Committee** | **University of Helsinki & Biobanks** | |
| Antti Hakanen | Auria Biobank / University of Turku / Hospital District of Southwest Finland, Turku, Finland | antti.hakanen@tyks.fi | **Steering Committee** | **University of Helsinki & Biobanks** | |
| Terhi Kilpi | THL Biobank / Finnish Institute for Health and Welfare (THL), Helsinki, Finland | terhi.kilpi@thl.fi | **Steering Committee** | **University of Helsinki & Biobanks** | |
| Markus Perola | THL Biobank / Finnish Institute for Health and Welfare (THL), Helsinki, Finland | markus.perola@thl.fi | **Steering Committee** | **University of Helsinki & Biobanks** | |
| Jukka Partanen | Finnish Red Cross Blood Service / Finnish Hematology Registry and Clinical Biobank, Helsinki, Finland | jukka.partanen@veripalvelu.fi | **Steering Committee** | **University of Helsinki & Biobanks** | |
| Taneli Raivio | Helsinki Biobank / Helsinki University and Hospital District of Helsinki and Uusimaa, Helsinki | taneli.raivio@hus.fi | **Steering Committee** | **University of Helsinki & Biobanks** | |
| Jani Tikkanen | Northern Finland Biobank Borealis / University of Oulu / Northern Ostrobothnia Hospital District, Oulu, Finland | jani.tikkanen@ppshp.fi | **Steering Committee** | **University of Helsinki & Biobanks** | |
| Raisa Serpi | Northern Finland Biobank Borealis / University of Oulu / Northern Ostrobothnia Hospital District, Oulu, Finland | raisa.serpi@ppshp.fi | **Steering Committee** | **University of Helsinki & Biobanks** | |
| Kati Kristiansson | Finnish Clinical Biobank Tampere / University of Tampere / Pirkanmaa Hospital District, Tampere, Finland | kati.kristiansson@pirha.fi | **Steering Committee** | **University of Helsinki & Biobanks** | |
| Veli-Matti Kosma | Biobank of Eastern Finland / University of Eastern Finland / Northern Savo Hospital District, Kuopio, Finland | veli-matti.kosma@uef.fi | **Steering Committee** | **University of Helsinki & Biobanks** | |
| Jari Laukkanen | Central Finland Biobank / University of Jyväskylä / Central Finland Health Care District, Jyväskylä, Finland | jari.laukkanen@ksshp.fi | **Steering Committee** | **University of Helsinki & Biobanks** | |
| Marco Hautalahti | FINBB - Finnish biobank cooperative | marco.hautalahti@finbb.fi | **Steering Committee** | **University of Helsinki & Biobanks** | |
| Outi Tuovila | Business Finland, Helsinki, Finland | outi.tuovila@businessfinland.fi | **Steering Committee** | **Other Experts/ Non-Voting Members** | |
| Jeffrey Waring | Abbvie, Chicago, IL, United States | jeff.waring@abbvie.com | **Scientific Committee** | **Pharmaceutical companies** | |
| Bridget Riley-Gillis | Abbvie, Chicago, IL, United States | bridget.rileygillis@abbvie.com | **Scientific Committee** | **Pharmaceutical companies** | |
| Fedik Rahimov | Abbvie, Chicago, IL, United States | fedik.rahimov@abbvie.com | **Scientific Committee** | **Pharmaceutical companies** | |
| Ioanna Tachmazidou | Astra Zeneca, Cambridge, United Kingdom | ioanna.tachmazidou@astrazeneca.com | **Scientific Committee** | **Pharmaceutical companies** | |
| Chia-Yen Chen | Biogen, Cambridge, MA, United States | chiayen.chen@biogen.com | **Scientific Committee** | **Pharmaceutical companies** | |
| Zhihao Ding | Boehringer Ingelheim, Ingelheim am Rhein, Germany | zhihao.ding@boehringer-ingelheim.com | **Scientific Committee** | **Pharmaceutical companies** | |
| Marc Jung | Boehringer Ingelheim, Ingelheim am Rhein, Germany | marc_oliver.jung@boehringer-ingelheim.com | **Scientific Committee** | **Pharmaceutical companies** | |
| Hanati Tuoken | Boehringer Ingelheim, Ingelheim am Rhein, Germany | hanati.tuoken@boehringer-ingelheim.com | **Scientific Committee** | **Pharmaceutical companies** | |
| Shameek Biswas | Bristol Myers Squibb, New York, NY, United States | Shameek.Biswas@bms.com | **Scientific Committee** | **Pharmaceutical companies** | |
| Rion Pendergrass | Genentech, San Francisco, CA, United States | penders2@gene.com | **Scientific Committee** | **Pharmaceutical companies** | |
| Jonathan Davitte | GlaxoSmithKline, Collegeville, PA, United States | jonathan.m.davitte@gsk.com | **Scientific Committee** | **Pharmaceutical companies** | |
| Neha Raghavan | Merck, Kenilworth, NJ, United States | neha.raghavan@merck.com | **Scientific Committee** | **Pharmaceutical companies** | |
| Adriana Huertas-Vazquez | Merck, Kenilworth, NJ, United States | adriana.huertas.vazquez@merck.com | **Scientific Committee** | **Pharmaceutical companies** | |
| Jae-Hoon Sul | Merck, Kenilworth, NJ, United States | jae.hoon.sul@merck.com | **Scientific Committee** | **Pharmaceutical companies** | |
| Anders Mälarstig | Pfizer, New York, NY, United States | anders.malarstig@pfizer.com | **Scientific Committee** | **Pharmaceutical companies** | |
| Xinli Hu | Pfizer, New York, NY, United States | xinli.hu@pfizer.com | **Scientific Committee** | **Pharmaceutical companies** | |
| Åsa Hedman | Pfizer, New York, NY, United States | asa.hedman@pfizer.com | **Scientific Committee** | **Pharmaceutical companies** | |
| Katherine Klinger | Translational Sciences, Sanofi R&D, Framingham, MA, USA | katherine.klinger@sanofi.com | **Scientific Committee** | **Pharmaceutical companies** | |
| Robert Graham | Maze Therapeutics, San Francisco, CA, United States | rgraham@mazetx.com | **Scientific Committee** | **Pharmaceutical companies** | |
| Dawn Waterworth | Janssen Research & Development, LLC, Spring House, PA, United States | dwaterwo@its.jnj.com | **Scientific Committee** | **Pharmaceutical companies** | |
| Nicole Renaud | Novartis Institutes for BioMedical Research, Cambridge, MA, United States | nicole.renaud@novartis.com | **Scientific Committee** | **Pharmaceutical companies** | |
| Ma´en Obeidat | Novartis Institutes for BioMedical Research, Cambridge, MA, United States | maen.obeidat@novartis.com | **Scientific Committee** | **Pharmaceutical companies** | |
| Jonathan Chung | Novartis Institutes for BioMedical Research, Cambridge, MA, United States | jonathan.chung@novartis.com | **Scientific Committee** | **Pharmaceutical companies** | |
| Jonas Zierer | Novartis Institutes for BioMedical Research, Cambridge, MA, United States | jonas.zierer@novartis.com | **Scientific Committee** | **Pharmaceutical companies** | |
| Mari Niemi | Novartis Institutes for BioMedical Research, Cambridge, MA, United States | mari.niemi@novartis.com | **Scientific Committee** | **Pharmaceutical companies** | |
| Samuli Ripatti | Institute for Molecular Medicine Finland (FIMM), HiLIFE, University of Helsinki, Helsinki, Finland | samuli.ripatti@helsinki.fi | **Scientific Committee** | **University of Helsinki & Biobanks** | |
| Johanna Schleutker | Auria Biobank / Univ. of Turku / Hospital District of Southwest Finland, Turku, Finland | johanna.schleutker@utu.fi | **Scientific Committee** | **University of Helsinki & Biobanks** | |
| Markus Perola | THL Biobank / Finnish Institute for Health and Welfare (THL), Helsinki, Finland | markus.perola@thl.fi | **Scientific Committee** | **University of Helsinki & Biobanks** | |
| Mikko Arvas | Finnish Red Cross Blood Service / Finnish Hematology Registry and Clinical Biobank, Helsinki, Finland | mikko.arvas@veripalvelu.fi | **Scientific Committee** | **University of Helsinki & Biobanks** | |
| Olli Carpén | Helsinki Biobank / Helsinki University and Hospital District of Helsinki and Uusimaa, Helsinki | olli.carpen@helsinki.fi | **Scientific Committee** | **University of Helsinki & Biobanks** | |
| Reetta Hinttala | Northern Finland Biobank Borealis / University of Oulu / Northern Ostrobothnia Hospital District, Oulu, Finland | reetta.hinttala@oulu.fi | **Scientific Committee** | **University of Helsinki & Biobanks** | |
| Johannes Kettunen | Northern Finland Biobank Borealis / University of Oulu / Northern Ostrobothnia Hospital District, Oulu, Finland | johannes.kettunen@oulu.fi | **Scientific Committee** | **University of Helsinki & Biobanks** | |
| Arto Mannermaa | Biobank of Eastern Finland / University of Eastern Finland / Northern Savo Hospital District, Kuopio, Finland | arto.mannermaa@uef.fi | **Scientific Committee** | **University of Helsinki & Biobanks** | |
| Katriina Aalto-Setälä | Faculty of Medicine and Health Technology, Tampere University, Tampere, Finland | katriina.aalto-setala@tuni.fi | **Scientific Committee** | **University of Helsinki & Biobanks** | |
| Mika Kähönen | Finnish Clinical Biobank Tampere / University of Tampere / Pirkanmaa Hospital District, Tampere, Finland | mika.kahonen@uta.fi | **Scientific Committee** | **University of Helsinki & Biobanks** | |
| Jari Laukkanen | Central Finland Biobank / University of Jyväskylä / Central Finland Health Care District, Jyväskylä, Finland | jari.laukkanen@ksshp.fi | **Scientific Committee** | **University of Helsinki & Biobanks** | |
| Johanna Mäkelä | FINBB - Finnish biobank cooperative | johanna.makela@finbb.fi | **Scientific Committee** | **University of Helsinki & Biobanks** | |
| Reetta Kälviäinen | Northern Savo Hospital District, Kuopio, Finland | reetta.kalviainen@kuh.fi | **Clinical Groups** | **Neurology Group** | |
| Valtteri Julkunen | Northern Savo Hospital District, Kuopio, Finland | valtteri.julkunen@kuh.fi | **Clinical Groups** | **Neurology Group** | |
| Hilkka Soininen | Northern Savo Hospital District, Kuopio, Finland | hilkka.soininen@uef.fi | **Clinical Groups** | **Neurology Group** | |
| Anne Remes | Northern Ostrobothnia Hospital District, Oulu, Finland | anne.remes@oulu.fi | **Clinical Groups** | **Neurology Group** | |
| Mikko Hiltunen | University of Eastern Finland, Kuopio, Finland | mikko.hiltunen@uef.fi | **Clinical Groups** | **Neurology Group** | |
| Jukka Peltola | Pirkanmaa Hospital District, Tampere, Finland | jukka.peltola@pshp.fi | **Clinical Groups** | **Neurology Group** | |
| Minna Raivio | Hospital District of Helsinki and Uusimaa, Helsinki, Finland | minna.raivio@geri.fi | **Clinical Groups** | **Neurology Group** | |
| Pentti Tienari | Hospital District of Helsinki and Uusimaa, Helsinki, Finland | pentti.tienari@hus.fi | **Clinical Groups** | **Neurology Group** | |
| Juha Rinne | Hospital District of Southwest Finland, Turku, Finland | juha.rinne@tyks.fi | **Clinical Groups** | **Neurology Group** | |
| Roosa Kallionpää | Hospital District of Southwest Finland, Turku, Finland | roosa.kallionpaa@tyks.fi | **Clinical Groups** | **Neurology Group** | |
| Juulia Partanen | Institute for Molecular Medicine Finland, HiLIFE, University of Helsinki, Finland | juulia.partanen@helsinki.fi | **Clinical Groups** | **Neurology Group** | |
| Adam Ziemann | Abbvie, Chicago, IL, United States | adam.ziemann@abbvie.com | **Clinical Groups** | **Neurology Group** | |
| Nizar Smaoui | Abbvie, Chicago, IL, United States | nizar.smaoui@abbvie.com | **Clinical Groups** | **Neurology Group** | |
| Anne Lehtonen | Abbvie, Chicago, IL, United States | anne.lehtonen@abbvie.com | **Clinical Groups** | **Neurology Group** | |
| Susan Eaton | Biogen, Cambridge, MA, United States | susan.eaton@biogen.com | **Clinical Groups** | **Neurology Group** | |
| Heiko Runz | Biogen, Cambridge, MA, United States | heiko.runz@biogen.com | **Clinical Groups** | **Neurology Group** | |
| Sanni Lahdenperä | Biogen, Cambridge, MA, United States | sanni.lahdenpera@biogen.com | **Clinical Groups** | **Neurology Group** | |
| Shameek Biswas | Bristol Myers Squibb, New York, NY, United States | shameek.biswas@bms.com | **Clinical Groups** | **Neurology Group** | |
| Natalie Bowers | Genentech, San Francisco, CA, United States | bowersn1@gene.com | **Clinical Groups** | **Neurology Group** | |
| Edmond Teng | Genentech, San Francisco, CA, United States | teng.edmond@gene.com | **Clinical Groups** | **Neurology Group** | |
| Rion Pendergrass | Genentech, San Francisco, CA, United States | penders2@gene.com | **Clinical Groups** | **Neurology Group** | |
| Fanli Xu | GlaxoSmithKline, Brentford, United Kingdom | chun-fang.2.xu@gsk.com | **Clinical Groups** | **Neurology Group** | |
| Kirsi Auro | GlaxoSmithKline, Espoo, Finland | kirsi.m.auro@gsk.com | **Clinical Groups** | **Neurology Group** | |
| Laura Addis | GlaxoSmithKline, Brentford, United Kingdom | laura.x.addis@gsk.com | **Clinical Groups** | **Neurology Group** | |
| John Eicher | GlaxoSmithKline, Brentford, United Kingdom | john.d.eicher@gsk.com | **Clinical Groups** | **Neurology Group** | |
| Qingqin S Li | Janssen Research & Development, LLC, Titusville, NJ 08560, United States | QLi2@its.jnj.com | **Clinical Groups** | **Neurology Group** | |
| Karen He | Janssen Research & Development, LLC, Spring House, PA, United States | khe2@its.jnj.com | **Clinical Groups** | **Neurology Group** | |
| Ekaterina Khramtsova | Janssen Research & Development, LLC, Spring House, PA, United States | ekhramts@its.jnj.com | **Clinical Groups** | **Neurology Group** | |
| Neha Raghavan | Merck, Kenilworth, NJ, United States | neha.raghavan@merck.com | **Clinical Groups** | **Neurology Group** | |
| Martti Färkkilä | Hospital District of Helsinki and Uusimaa, Helsinki, Finland | martti.farkkila@hus.fi | **Clinical Groups** | **Gastroenterology Group** | |
| Jukka Koskela | Hospital District of Helsinki and Uusimaa, Helsinki, Finland | jukka.koskela@helsinki.fi | **Clinical Groups** | **Gastroenterology Group** | |
| Sampsa Pikkarainen | Hospital District of Helsinki and Uusimaa, Helsinki, Finland | sampsa.pikkarainen@hus.fi | **Clinical Groups** | **Gastroenterology Group** | |
| Airi Jussila | Pirkanmaa Hospital District, Tampere, Finland | airi.jussila@pshp.fi | **Clinical Groups** | **Gastroenterology Group** | |
| Katri Kaukinen | Pirkanmaa Hospital District, Tampere, Finland | katri.kaukinen@tuni.fi | **Clinical Groups** | **Gastroenterology Group** | |
| Timo Blomster | Northern Ostrobothnia Hospital District, Oulu, Finland | timo.blomster@ppshp.fi | **Clinical Groups** | **Gastroenterology Group** | |
| Mikko Kiviniemi | Northern Savo Hospital District, Kuopio, Finland | mikko.kiviniemi@kuh.fi | **Clinical Groups** | **Gastroenterology Group** | |
| Markku Voutilainen | Hospital District of Southwest Finland, Turku, Finland | markku.voutilainen@tyks.fi | **Clinical Groups** | **Gastroenterology Group** | |
| Mark Daly | Institute for Molecular Medicine, Finland (FIMM), HiLIFE, University of Helsinki, Helsinki, Finland; Broad Institute of MIT and Harvard; Massachusetts General Hospital | mark.daly@helsinki.fi | **Clinical Groups** | **Gastroenterology Group** | |
| Jeffrey Waring | Abbvie, Chicago, IL, United States | jeff.waring@abbvie.com | **Clinical Groups** | **Gastroenterology Group** | |
| Nizar Smaoui | Abbvie, Chicago, IL, United States | nizar.smaoui@abbvie.com | **Clinical Groups** | **Gastroenterology Group** | |
| Fedik Rahimov | Abbvie, Chicago, IL, United States | fedik.rahimov@abbvie.com | **Clinical Groups** | **Gastroenterology Group** | |
| Anne Lehtonen | Abbvie, Chicago, IL, United States | anne.lehtonen@abbvie.com | **Clinical Groups** | **Gastroenterology Group** | |
| Tim Lu | Genentech, San Francisco, CA, United States | lut8@gene.com | **Clinical Groups** | **Gastroenterology Group** | |
| Natalie Bowers | Genentech, San Francisco, CA, United States | bowersn1@gene.com | **Clinical Groups** | **Gastroenterology Group** | |
| Rion Pendergrass | Genentech, San Francisco, CA, United States | penders2@gene.com | **Clinical Groups** | **Gastroenterology Group** | |
| Linda McCarthy | GlaxoSmithKline, Brentford, United Kingdom | linda.c.mccarthy@gsk.com | **Clinical Groups** | **Gastroenterology Group** | |
| Amy Hart | Janssen Research & Development, LLC, Spring House, PA, United States | ahart13@its.jnj.com | **Clinical Groups** | **Gastroenterology Group** | |
| Meijian Guan | Janssen Research & Development, LLC, Spring House, PA, United States | mguan4@its.jnj.com | **Clinical Groups** | **Gastroenterology Group** | |
| Jason Miller | Merck, Kenilworth, NJ, United States | jason.miller4@merck.com | **Clinical Groups** | **Gastroenterology Group** | |
| Kirsi Kalpala | Pfizer, New York, NY, United States | kirsi.kalpala@pfizer.com | **Clinical Groups** | **Gastroenterology Group** | |
| Melissa Miller | Pfizer, New York, NY, United States | melissa.r.miller@pfizer.com | **Clinical Groups** | **Gastroenterology Group** | |
| Xinli Hu | Pfizer, New York, NY, United States | xinli.hu@pfizer.com | **Clinical Groups** | **Gastroenterology Group** | |
| Kari Eklund | Hospital District of Helsinki and Uusimaa, Helsinki, Finland | kari.eklund@hus.fi | **Clinical Groups** | **Rheumatology Group** | |
| Antti Palomäki | Hospital District of Southwest Finland, Turku, Finland | ajpalo@utu.fi | **Clinical Groups** | **Rheumatology Group** | |
| Pia Isomäki | Pirkanmaa Hospital District, Tampere, Finland | pia.isomaki@pshp.fi | **Clinical Groups** | **Rheumatology Group** | |
| Laura Pirilä | Hospital District of Southwest Finland, Turku, Finland | laura.pirila@fimnet.fi,laura.pirila@tyks.fi | **Clinical Groups** | **Rheumatology Group** | |
| Oili Kaipiainen-Seppänen | Northern Savo Hospital District, Kuopio, Finland | oili.kaipiainen-seppanen@kuh.fi | **Clinical Groups** | **Rheumatology Group** | |
| Johanna Huhtakangas | Northern Ostrobothnia Hospital District, Oulu, Finland | johanna.huhtakangas@kuh.fi | **Clinical Groups** | **Rheumatology Group** | |
| Nina Mars | Institute for Molecular Medicine Finland (FIMM), HiLIFE, University of Helsinki, Helsinki, Finland | nina.mars@helsinki.fi | **Clinical Groups** | **Rheumatology Group** | |
| Jeffrey Waring | Abbvie, Chicago, IL, United States | jeff.waring@abbvie.com | **Clinical Groups** | **Rheumatology Group** | |
| Fedik Rahimov | Abbvie, Chicago, IL, United States | fedik.rahimov@abbvie.com | **Clinical Groups** | **Rheumatology Group** | |
| Apinya Lertratanakul | Abbvie, Chicago, IL, United States | apinya.lertratanakul@abbvie.com | **Clinical Groups** | **Rheumatology Group** | |
| Nizar Smaoui | Abbvie, Chicago, IL, United States | nizar.smaoui@abbvie.com | **Clinical Groups** | **Rheumatology Group** | |
| Anne Lehtonen | Abbvie, Chicago, IL, United States | anne.lehtonen@abbvie.com | **Clinical Groups** | **Rheumatology Group** | |
| Coralie Viollet | AstraZeneca, Cambridge, United Kingdom | coralie.viollet@astrazeneca.com | **Clinical Groups** | **Rheumatology Group** | |
| Marla Hochfeld | Bristol Myers Squibb, New York, NY, United States | mhochfeld@celgene.com | **Clinical Groups** | **Rheumatology Group** | |
| Natalie Bowers | Genentech, San Francisco, CA, United States | bowersn1@gene.com | **Clinical Groups** | **Rheumatology Group** | |
| Rion Pendergrass | Genentech, San Francisco, CA, United States | penders2@gene.com | **Clinical Groups** | **Rheumatology Group** | |
| Jorge Esparza Gordillo | GlaxoSmithKline, Brentford, United Kingdom | jorge.x.esparza-gordillo@gsk.com | **Clinical Groups** | **Rheumatology Group** | |
| Kirsi Auro | GlaxoSmithKline, Espoo, Finland | kirsi.m.auro@gsk.com | **Clinical Groups** | **Rheumatology Group** | |
| Dawn Waterworth | Janssen Research & Development, LLC, Spring House, PA, United States | dwaterwo@its.jnj.com | **Clinical Groups** | **Rheumatology Group** | |
| Fabiana Farias | Merck, Kenilworth, NJ, United States | fabiana.farias@merck.com | **Clinical Groups** | **Rheumatology Group** | |
| Kirsi Kalpala | Pfizer, New York, NY, United States | kirsi.kalpala@pfizer.com | **Clinical Groups** | **Rheumatology Group** | |
| Nan Bing | Pfizer, New York, NY, United States | nan.bing@pfizer.com | **Clinical Groups** | **Rheumatology Group** | |
| Xinli Hu | Pfizer, New York, NY, United States | xinli.hu@pfizer.com | **Clinical Groups** | **Rheumatology Group** | |
| Tarja Laitinen | Pirkanmaa Hospital District, Tampere, Finland | tarja.laitinen@pshp.fi | **Clinical Groups** | **Pulmonology Group** | |
| Margit Pelkonen | Northern Savo Hospital District, Kuopio, Finland | margit.pelkonen@kuh.fi | **Clinical Groups** | **Pulmonology Group** | |
| Paula Kauppi | Hospital District of Helsinki and Uusimaa, Helsinki, Finland | paula.kauppi@hus.fi | **Clinical Groups** | **Pulmonology Group** | |
| Hannu Kankaanranta | University of Gothenburg, Gothenburg, Sweden/ Seinäjoki Central Hospital, Seinäjoki, Finland/ Tampere University, Tampere, Finland | hannu.kankaanranta@tuni.fi | **Clinical Groups** | **Pulmonology Group** | |
| Terttu Harju | Northern Ostrobothnia Hospital District, Oulu, Finland | terttu.harju@oulu.fi | **Clinical Groups** | **Pulmonology Group** | |
| Riitta Lahesmaa | Hospital District of Southwest Finland, Turku, Finland | rilahes@utu.fi | **Clinical Groups** | **Pulmonology Group** | |
| Nizar Smaoui | Abbvie, Chicago, IL, United States | nizar.smaoui@abbvie.com | **Clinical Groups** | **Pulmonology Group** | |
| Coralie Viollet | AstraZeneca, Cambridge, United Kingdom | coralie.viollet@astrazeneca.com | **Clinical Groups** | **Pulmonology Group** | |
| Susan Eaton | Biogen, Cambridge, MA, United States | susan.eaton@biogen.com | **Clinical Groups** | **Pulmonology Group** | |
| Hubert Chen | Genentech, San Francisco, CA, United States | chenh37@gene.com | **Clinical Groups** | **Pulmonology Group** | |
| Rion Pendergrass | Genentech, San Francisco, CA, United States | penders2@gene.com | **Clinical Groups** | **Pulmonology Group** | |
| Natalie Bowers | Genentech, San Francisco, CA, United States | bowersn1@gene.com | **Clinical Groups** | **Pulmonology Group** | |
| Joanna Betts | GlaxoSmithKline, Brentford, United Kingdom | joanna.c.betts@gsk.com | **Clinical Groups** | **Pulmonology Group** | |
| Kirsi Auro | GlaxoSmithKline, Espoo, Finland | kirsi.m.auro@gsk.com | **Clinical Groups** | **Pulmonology Group** | |
| Rajashree Mishra | GlaxoSmithKline, Brentford, United Kingdom | rajashree.x.mishra@gsk.com | **Clinical Groups** | **Pulmonology Group** | |
| Majd Mouded | Novartis, Basel, Switzerland | majd.mouded@novartis.com | **Clinical Groups** | **Pulmonology Group** | |
| Debby Ngo | Novartis, Basel, Switzerland | debby.ngo@novartis.com | **Clinical Groups** | **Pulmonology Group** | |
| Teemu Niiranen | Finnish Institute for Health and Welfare (THL), Helsinki, Finland | teemu.niiranen@thl.fi | **Clinical Groups** | **Cardiometabolic Diseases Group** | |
| Felix Vaura | Finnish Institute for Health and Welfare (THL), Helsinki, Finland | fechva@utu.fi | **Clinical Groups** | **Cardiometabolic Diseases Group** | |
| Veikko Salomaa | Finnish Institute for Health and Welfare (THL), Helsinki, Finland | veikko.salomaa@thl.fi | **Clinical Groups** | **Cardiometabolic Diseases Group** | |
| Kaj Metsärinne | Hospital District of Southwest Finland, Turku, Finland | kaj.metsarinne@tyks.fi | **Clinical Groups** | **Cardiometabolic Diseases Group** | |
| Jenni Aittokallio | Hospital District of Southwest Finland, Turku, Finland | jemato@utu.fi | **Clinical Groups** | **Cardiometabolic Diseases Group** | |
| Mika Kähönen | Pirkanmaa Hospital District, Tampere, Finland | mika.kahonen@uta.fi | **Clinical Groups** | **Cardiometabolic Diseases Group** | |
| Jussi Hernesniemi | Pirkanmaa Hospital District, Tampere, Finland | jussi.hernesniemi@tuni.fi | **Clinical Groups** | **Cardiometabolic Diseases Group** | |
| Daniel Gordin | Hospital District of Helsinki and Uusimaa, Helsinki, Finland | daniel.gordin@hus.fi | **Clinical Groups** | **Cardiometabolic Diseases Group** | |
| Juha Sinisalo | Hospital District of Helsinki and Uusimaa, Helsinki, Finland | juha.sinisalo@hus.fi | **Clinical Groups** | **Cardiometabolic Diseases Group** | |
| Marja-Riitta Taskinen | Hospital District of Helsinki and Uusimaa, Helsinki, Finland | marja-riitta.taskinen@helsinki.fi | **Clinical Groups** | **Cardiometabolic Diseases Group** | |
| Tiinamaija Tuomi | Hospital District of Helsinki and Uusimaa, Helsinki, Finland | tiinamaija.tuomi@hus.fi | **Clinical Groups** | **Cardiometabolic Diseases Group** | |
| Timo Hiltunen | Hospital District of Helsinki and Uusimaa, Helsinki, Finland | timo.hiltunen@hus.fi | **Clinical Groups** | **Cardiometabolic Diseases Group** | |
| Jari Laukkanen | Central Finland Health Care District, Jyväskylä, Finland | jari.laukkanen@ksshp.fi | **Clinical Groups** | **Cardiometabolic Diseases Group** | |
| Amanda Elliott | Institute for Molecular Medicine Finland (FIMM), HiLIFE, University of Helsinki, Helsinki, Finland; Broad Institute, Cambridge, MA, USA and Massachusetts General Hospital, Boston, MA, USA | aelliott@broadinstitute.org | **Clinical Groups** | **Cardiometabolic Diseases Group** | |
| Mary Pat Reeve | Institute for Molecular Medicine Finland (FIMM), HiLIFE, University of Helsinki, Helsinki, Finland | mary.reeve@helsinki.fi | **Clinical Groups** | **Cardiometabolic Diseases Group** | |
| Sanni Ruotsalainen | Institute for Molecular Medicine Finland (FIMM), HiLIFE, University of Helsinki, Helsinki, Finland | sanni.ruotsalainen@helsinki.fi | **Clinical Groups** | **Cardiometabolic Diseases Group** | |
| Dirk Paul | Astra Zeneca, Cambridge, United Kingdom | dirk.paul@astrazeneca.com | **Clinical Groups** | **Cardiometabolic Diseases Group** | |
| Natalie Bowers | Genentech, San Francisco, CA, United States | bowersn1@gene.com | **Clinical Groups** | **Cardiometabolic Diseases Group** | |
| Rion Pendergrass | Genentech, San Francisco, CA, United States | penders2@gene.com | **Clinical Groups** | **Cardiometabolic Diseases Group** | |
| Audrey Chu | GlaxoSmithKline, Brentford, United Kingdom | audrey.y.chu@gsk.com | **Clinical Groups** | **Cardiometabolic Diseases Group** | |
| Kirsi Auro | GlaxoSmithKline, Espoo, Finland | kirsi.m.auro@gsk.com | **Clinical Groups** | **Cardiometabolic Diseases Group** | |
| Dermot Reilly | Janssen Research & Development, LLC, Boston, MA, United States | dreill11@its.jnj.com | **Clinical Groups** | **Cardiometabolic Diseases Group** | |
| Mike Mendelson | Novartis, Boston, MA, United States | mike.mendelson@novartis.com | **Clinical Groups** | **Cardiometabolic Diseases Group** | |
| Jaakko Parkkinen | Pfizer, New York, NY, United States | jaakko.parkkinen@pfizer.com | **Clinical Groups** | **Cardiometabolic Diseases Group** | |
| Melissa Miller | Pfizer, New York, NY, United States | melissa.r.miller@pfizer.com | **Clinical Groups** | **Cardiometabolic Diseases Group** | |
| Tuomo Meretoja | Department of Breast Surgery, Helsinki University Hospital Comprehensive Cancer Center and University of Helsinki, Helsinki, Finland | tuomo.meretoja@hus.fi | **Clinical Groups** | **Oncology Group** | |
| Heikki Joensuu | Department of Oncology, Helsinki University Hospital Comprehensive Cancer Center and University of Helsinki, Helsinki, Finland | heikki.joensuu@hus.fi | **Clinical Groups** | **Oncology Group** | |
| Olli Carpén | Hospital District of Helsinki and Uusimaa, Helsinki, Finland | olli.carpen@helsinki.fi | **Clinical Groups** | **Oncology Group** | |
| Johanna Mattson | Hospital District of Helsinki and Uusimaa, Helsinki, Finland | johanna.mattson@hus.fi | **Clinical Groups** | **Oncology Group** | |
| Eveliina Salminen | Hospital District of Helsinki and Uusimaa, Helsinki, Finland | eveliina.e.salminen@hus.fi | **Clinical Groups** | **Oncology Group** | |
| Annika Auranen | Pirkanmaa Hospital District , Tampere, Finland | anaura@utu.fi | **Clinical Groups** | **Oncology Group** | |
| Peeter Karihtala | Department of Oncology, Helsinki University Hospital Comprehensive Cancer Center and University of Helsinki, Helsinki, Finland | peeter.karihtala@hus.fi | **Clinical Groups** | **Oncology Group** | |
| Päivi Auvinen | Northern Savo Hospital District, Kuopio, Finland | paivi.auvinen@kuh.fi | **Clinical Groups** | **Oncology Group** | |
| Klaus Elenius | Hospital District of Southwest Finland, Turku, Finland | klaus.elenius@utu.fi | **Clinical Groups** | **Oncology Group** | |
| Johanna Schleutker | Hospital District of Southwest Finland, Turku, Finland | johanna.schleutker@utu.fi | **Clinical Groups** | **Oncology Group** | |
| Esa Pitkänen | Institute for Molecular Medicine Finland (FIMM), HiLIFE, University of Helsinki, Helsinki, Finland | esa.pitkanen@helsinki.fi | **Clinical Groups** | **Oncology Group** | |
| Nina Mars | Institute for Molecular Medicine Finland (FIMM), HiLIFE, University of Helsinki, Helsinki, Finland | nina.mars@helsinki.fi | **Clinical Groups** | **Oncology Group** | |
| Mark Daly | Institute for Molecular Medicine Finland (FIMM), HiLIFE, University of Helsinki, Helsinki, Finland; Broad Institute of MIT and Harvard; Massachusetts General Hospital | mark.daly@helsinki.fi | **Clinical Groups** | **Oncology Group** | |
| Relja Popovic | Abbvie, Chicago, IL, United States | relja.popovic@abbvie.com | **Clinical Groups** | **Oncology Group** | |
| Jeffrey Waring | Abbvie, Chicago, IL, United States | jeff.waring@abbvie.com | **Clinical Groups** | **Oncology Group** | |
| Bridget Riley-Gillis | Abbvie, Chicago, IL, United States | bridget.rileygillis@abbvie.com | **Clinical Groups** | **Oncology Group** | |
| Anne Lehtonen | Abbvie, Chicago, IL, United States | anne.lehtonen@abbvie.com | **Clinical Groups** | **Oncology Group** | |
| Margarete Fabre | AstraZeneca, Cambridge, United Kingdom | margarete.fabre@astrazeneca.com | **Clinical Groups** | **Oncology Group** | |
| Jennifer Schutzman | Genentech, San Francisco, CA, United States | schutzman.jennifer@gene.com | **Clinical Groups** | **Oncology Group** | |
| Natalie Bowers | Genentech, San Francisco, CA, United States | bowersn1@gene.com | **Clinical Groups** | **Oncology Group** | |
| Rion Pendergrass | Genentech, San Francisco, CA, United States | penders2@gene.com | **Clinical Groups** | **Oncology Group** | |
| Diptee Kulkarni | GlaxoSmithKline, Brentford, United Kingdom | diptee.a.kulkarni@gsk.com | **Clinical Groups** | **Oncology Group** | |
| Kirsi Auro | GlaxoSmithKline, Espoo, Finland | kirsi.m.auro@gsk.com | **Clinical Groups** | **Oncology Group** | |
| Alessandro Porello | Janssen Research & Development, LLC, Spring House, PA, United States | APorrell@ITS.JNJ.com | **Clinical Groups** | **Oncology Group** | |
| Andrey Loboda | Merck, Kenilworth, NJ, United States | andrey_loboda@merck.com | **Clinical Groups** | **Oncology Group** | |
| Heli Lehtonen | Pfizer, New York, NY, United States | heli.lehtonen@pfizer.com | **Clinical Groups** | **Oncology Group** | |
| Stefan McDonough | Pfizer, New York, NY, United States | stefan.McDonough@pfizer.com | **Clinical Groups** | **Oncology Group** | |
| Sauli Vuoti | Janssen-Cilag Oy, Espoo, Finland | svuoti@its.jnj.com | **Clinical Groups** | **Oncology Group** | |
| Kai Kaarniranta | Northern Savo Hospital District, Kuopio, Finland; Department of Molecular Genetics, University of Lodz, Lodz, Poland | kai.kaarniranta@uef.fi | **Clinical Groups** | **Opthalmology Group** | |
| Joni A Turunen | Helsinki University Hospital and University of Helsinki, Helsinki, Finland; Eye Genetics Group, Folkhälsan Research Center, Helsinki, Finland | joni.turunen@helsinki.fi | **Clinical Groups** | **Opthalmology Group** | |
| Terhi Ollila | Hospital District of Helsinki and Uusimaa, Helsinki, Finland | terhi.ollila@hus.fi | **Clinical Groups** | **Opthalmology Group** | |
| Hannu Uusitalo | Pirkanmaa Hospital District, Tampere, Finland | hannu.uusitalo@tuni.fi | **Clinical Groups** | **Opthalmology Group** | |
| Juha Karjalainen | Institute for Molecular Medicine Finland (FIMM), HiLIFE, University of Helsinki, Helsinki, Finland | juha.karjalainen@helsinki.fi | **Clinical Groups** | **Opthalmology Group** | |
| Esa Pitkänen | Institute for Molecular Medicine Finland (FIMM), HiLIFE, University of Helsinki, Helsinki, Finland | esa.pitkanen@helsinki.fi | **Clinical Groups** | **Opthalmology Group** | |
| Mengzhen Liu | Abbvie, Chicago, IL, United States | mengzhen.liu@abbvie.com | **Clinical Groups** | **Opthalmology Group** | |
| Heiko Runz | Biogen, Cambridge, MA, United States | heiko.runz@biogen.com | **Clinical Groups** | **Opthalmology Group** | |
| Stephanie Loomis | Biogen, Cambridge, MA, United States | stephanie.loomis@biogen.com | **Clinical Groups** | **Opthalmology Group** | |
| Erich Strauss | Genentech, San Francisco, CA, United States | strauss.erich@gene.com | **Clinical Groups** | **Opthalmology Group** | |
| Natalie Bowers | Genentech, San Francisco, CA, United States | bowersn1@gene.com | **Clinical Groups** | **Opthalmology Group** | |
| Hao Chen | Genentech, San Francisco, CA, United States | haoc@gene.com | **Clinical Groups** | **Opthalmology Group** | |
| Rion Pendergrass | Genentech, San Francisco, CA, United States | penders2@gene.com | **Clinical Groups** | **Opthalmology Group** | |
| Kaisa Tasanen | Northern Ostrobothnia Hospital District, Oulu, Finland | kaisa.tasanen-maatta@oulu.fi | **Clinical Groups** | **Dermatology Group** | |
| Laura Huilaja | Northern Ostrobothnia Hospital District, Oulu, Finland | laura.huilaja@oulu.fi | **Clinical Groups** | **Dermatology Group** | |
| Katariina Hannula-Jouppi | Hospital District of Helsinki and Uusimaa, Helsinki, Finland | katariina.hannula-jouppi@hus.fi | **Clinical Groups** | **Dermatology Group** | |
| Teea Salmi | Pirkanmaa Hospital District, Tampere, Finland | teea.salmi@pshp.fi | **Clinical Groups** | **Dermatology Group** | |
| Sirkku Peltonen | Hospital District of Southwest Finland, Turku, Finland | sipelto@utu.fi | **Clinical Groups** | **Dermatology Group** | |
| Leena Koulu | Hospital District of Southwest Finland, Turku, Finland | leena.koulu@tyks.fi | **Clinical Groups** | **Dermatology Group** | |
| Nizar Smaoui | Abbvie, Chicago, IL, United States | nizar.smaoui@abbvie.com | **Clinical Groups** | **Dermatology Group** | |
| Fedik Rahimov | Abbvie, Chicago, IL, United States | fedik.rahimov@abbvie.com | **Clinical Groups** | **Dermatology Group** | |
| Anne Lehtonen | Abbvie, Chicago, IL, United States | anne.lehtonen@abbvie.com | **Clinical Groups** | **Dermatology Group** | |
| David Choy | Genentech, San Francisco, CA, United States | choy.david@gene.com | **Clinical Groups** | **Dermatology Group** | |
| Rion Pendergrass | Genentech, San Francisco, CA, United States | penders2@gene.com | **Clinical Groups** | **Dermatology Group** | |
| Dawn Waterworth | Janssen Research & Development, LLC, Spring House, PA, United States | dwaterwo@its.jnj.com | **Clinical Groups** | **Dermatology Group** | |
| Kirsi Kalpala | Pfizer, New York, NY, United States | kirsi.kalpala@pfizer.com | **Clinical Groups** | **Dermatology Group** | |
| Ying Wu | Pfizer, New York, NY, United States | ying.wu3@pfizer.com | **Clinical Groups** | **Dermatology Group** | |
| Pirkko Pussinen | Hospital District of Helsinki and Uusimaa, Helsinki, Finland | pirkko.pussinen@helsinki.fi | **Clinical Groups** | **Odontology Group** | |
| Aino Salminen | Hospital District of Helsinki and Uusimaa, Helsinki, Finland | aino.m.salminen@helsinki.fi | **Clinical Groups** | **Odontology Group** | |
| Tuula Salo | Hospital District of Helsinki and Uusimaa, Helsinki, Finland | tuula.salo@helsinki.fi | **Clinical Groups** | **Odontology Group** | |
| David Rice | Hospital District of Helsinki and Uusimaa, Helsinki, Finland | david.rice@helsinki.fi | **Clinical Groups** | **Odontology Group** | |
| Pekka Nieminen | Hospital District of Helsinki and Uusimaa, Helsinki, Finland | pekka.nieminen@helsinki.fi | **Clinical Groups** | **Odontology Group** | |
| Ulla Palotie | Hospital District of Helsinki and Uusimaa, Helsinki, Finland | ulla.palotie@helsinki.fi | **Clinical Groups** | **Odontology Group** | |
| Maria Siponen | Northern Savo Hospital District, Kuopio, Finland | maria.siponen@uef.fi | **Clinical Groups** | **Odontology Group** | |
| Liisa Suominen | Northern Savo Hospital District, Kuopio, Finland | liisa.suominen@uef.fi | **Clinical Groups** | **Odontology Group** | |
| Päivi Mäntylä | Northern Savo Hospital District, Kuopio, Finland | paivi.mantyla@uef.fi | **Clinical Groups** | **Odontology Group** | |
| Ulvi Gursoy | Hospital District of Southwest Finland, Turku, Finland | ulvi.gursoy@utu.fi | **Clinical Groups** | **Odontology Group** | |
| Vuokko Anttonen | Northern Ostrobothnia Hospital District, Oulu, Finland | vuokko.anttonen@oulu.fi | **Clinical Groups** | **Odontology Group** | |
| Kirsi Sipilä | Research Unit of Oral Health Sciences Faculty of Medicine, University of Oulu, Oulu, Finland; Medical Research Center, Oulu, Oulu University Hospital and University of Oulu, Oulu, Finland | kirsi.sipila@oulu.fi | **Clinical Groups** | **Odontology Group** | |
| Rion Pendergrass | Genentech, San Francisco, CA, United States | pendergrass.sarah@gene.com | **Clinical Groups** | **Odontology Group** | |
| Hannele Laivuori | Institute for Molecular Medicine Finland (FIMM), HiLIFE, University of Helsinki, Helsinki, Finland | hannele.laivuori@helsinki.fi | **Clinical Groups** | **Women’s Health and Reproduction Group** | |
| Venla Kurra | Pirkanmaa Hospital District, Tampere, Finland | venla.kurra@tuni.fi | **Clinical Groups** | **Women’s Health and Reproduction Group** | |
| Laura Kotaniemi-Talonen | Pirkanmaa Hospital District, Tampere, Finland | laura.kotaniemi-talonen@tuni.fi | **Clinical Groups** | **Women’s Health and Reproduction Group** | |
| Oskari Heikinheimo | Hospital District of Helsinki and Uusimaa, Helsinki, Finland | oskari.heikinheimo@helsinki.fi | **Clinical Groups** | **Women’s Health and Reproduction Group** | |
| Ilkka Kalliala | Hospital District of Helsinki and Uusimaa, Helsinki, Finland | ilkka.kalliala@hus.fi | **Clinical Groups** | **Women’s Health and Reproduction Group** | |
| Lauri Aaltonen | Hospital District of Helsinki and Uusimaa, Helsinki, Finland | lauri.aaltonen@helsinki.fi | **Clinical Groups** | **Women’s Health and Reproduction Group** | |
| Varpu Jokimaa | Hospital District of Southwest Finland, Turku, Finland | varpu.jokimaa@utu.fi | **Clinical Groups** | **Women’s Health and Reproduction Group** | |
| Johannes Kettunen | Northern Ostrobothnia Hospital District, Oulu, Finland | Johannes.Kettunen@oulu.fi | **Clinical Groups** | **Women’s Health and Reproduction Group** | |
| Marja Vääräsmäki | Northern Ostrobothnia Hospital District, Oulu, Finland | marja.vaarasmaki@oulu.fi | **Clinical Groups** | **Women’s Health and Reproduction Group** | |
| Outi Uimari | Northern Ostrobothnia Hospital District, Oulu, Finland | outi.uimari@oulu.fi | **Clinical Groups** | **Women’s Health and Reproduction Group** | |
| Laure Morin-Papunen | Northern Ostrobothnia Hospital District, Oulu, Finland | lmp@cc.oulu.fi | **Clinical Groups** | **Women’s Health and Reproduction Group** | |
| Maarit Niinimäki | Northern Ostrobothnia Hospital District, Oulu, Finland | maarit.niinimaki@oulu.fi | **Clinical Groups** | **Women’s Health and Reproduction Group** | |
| Terhi Piltonen | Northern Ostrobothnia Hospital District, Oulu, Finland | terhi.piltonen@oulu.fi | **Clinical Groups** | **Women’s Health and Reproduction Group** | |
| Katja Kivinen | Institute for Molecular Medicine Finland (FIMM), HiLIFE, University of Helsinki, Helsinki, Finland | katja.kivinen@helsinki.fi | **Clinical Groups** | **Women’s Health and Reproduction Group** | |
| Elisabeth Widen | Institute for Molecular Medicine Finland (FIMM), HiLIFE, University of Helsinki, Helsinki, Finland | elisabeth.widen@helsinki.fi | **Clinical Groups** | **Women’s Health and Reproduction Group** | |
| Taru Tukiainen | Institute for Molecular Medicine Finland (FIMM), HiLIFE, University of Helsinki, Helsinki, Finland | taru.tukiainen@helsinki.fi | **Clinical Groups** | **Women’s Health and Reproduction Group** | |
| Mary Pat Reeve | Institute for Molecular Medicine Finland (FIMM), HiLIFE, University of Helsinki, Helsinki, Finland | mary.reeve@helsinki.fi | **Clinical Groups** | **Women’s Health and Reproduction Group** | |
| Mark Daly | Institute for Molecular Medicine Finland (FIMM), HiLIFE, University of Helsinki, Helsinki, Finland; Broad Institute of MIT and Harvard; Massachusetts General Hospital | mark.daly@helsinki.fi | **Clinical Groups** | **Women’s Health and Reproduction Group** | |
| Niko Välimäki | University of Helsinki, Helsinki, Finland | niko.valimaki@helsinki.fi | **Clinical Groups** | **Women’s Health and Reproduction Group** | |
| Eija Laakkonen | University of Jyväskylä, Jyväskylä, Finland | eija.k.laakkonen@jyu.fi | **Clinical Groups** | **Women’s Health and Reproduction Group** | |
| Jaakko Tyrmi | University of Oulu, Oulu, Finland / University of Tampere, Tampere, Finland | jaakko.tyrmi@oulu.fi | **Clinical Groups** | **Women’s Health and Reproduction Group** | |
| Heidi Silven | University of Oulu, Oulu, Finland | heidi.silven@student.oulu.fi | **Clinical Groups** | **Women’s Health and Reproduction Group** | |
| Eeva Sliz | University of Oulu, Oulu, Finland | eeva.sliz@oulu.fi | **Clinical Groups** | **Women’s Health and Reproduction Group** | |
| Riikka Arffman | University of Oulu, Oulu, Finland | riikka.arffman@oulu.fi | **Clinical Groups** | **Women’s Health and Reproduction Group** | |
| Susanna Savukoski | University of Oulu, Oulu, Finland | susanna.savukoski@oulu.fi | **Clinical Groups** | **Women’s Health and Reproduction Group** | |
| Triin Laisk | Estonian biobank, Tartu, Estonia | triin.laisk@ut.ee | **Clinical Groups** | **Women’s Health and Reproduction Group** | |
| Natalia Pujol | Estonian biobank, Tartu, Estonia | natalia.pujolgualdo@oulu.fi | **Clinical Groups** | **Women’s Health and Reproduction Group** | |
| Mengzhen Liu | Abbvie, Chicago, IL, United States | mengzhen.liu@abbvie.com | **Clinical Groups** | **Women’s Health and Reproduction Group** | |
| Bridget Riley-Gillis | Abbvie, Chicago, IL, United States | bridget.rileygillis@abbvie.com | **Clinical Groups** | **Women’s Health and Reproduction Group** | |
| Rion Pendergrass | Genentech, San Francisco, CA, United States | penders2@gene.com | **Clinical Groups** | **Women’s Health and Reproduction Group** | |
| Janet Kumar | GlaxoSmithKline, Collegeville, PA, United States | janet.x.kumar@gsk.com | **Clinical Groups** | **Women’s Health and Reproduction Group** | |
| Kirsi Auro | GlaxoSmithKline, Espoo, Finland | kirsi.m.auro@gsk.com | **Clinical Groups** | **Women’s Health and Reproduction Group** | |
| Iiris Hovatta | University of Helsinki, Finland | iiris.hovatta@helsinki.fi | **Clinical Groups** | **Depression group** | |
| Chia-Yen Chen | Biogen, Cambridge, MA, United States | chiayen.chen@biogen.com | **Clinical Groups** | **Depression group** | |
| Erkki Isometsä | Hospital District of Helsinki and Uusimaa, Helsinki, Finland | erkki.isometsa@hus.fi | **Clinical Groups** | **Depression group** | |
| Hanna Ollila | Institute for Molecular Medicine Finland (FIMM), HiLIFE, University of Helsinki, Helsinki, Finland | hanna.m.ollila@helsinki.fi | **Clinical Groups** | **Depression group** | |
| Jaana Suvisaari | Finnish Institute for Health and Welfare (THL), Helsinki, Finland | jaana.suvisaari@thl.fi | **Clinical Groups** | **Depression group** | |
| Antti Mäkitie | Department of Otorhinolaryngology - Head and Neck Surgery, University of Helsinki and Helsinki University Hospital, Helsinki, Finland | antti.makitie@helsinki.fi | **Clinical Groups** | **ENT (ear, nose and throath) Group** | |
| Argyro Bizaki-Vallaskangas | Pirkanmaa Hospital District, Tampere, Finland | argyro.bizaki-vallaskangas@tuni.fi | **Clinical Groups** | **ENT (ear, nose and throath) Group** | |
| Sanna Toppila-Salmi | University of Eastern Finland and Kuopio University Hospital, Department of Otorhinolaryngology, Kuopio, Finland and Department of Allergy, Helsinki University Hospital and University of Helsinki, Finland | sanna.salmi@helsinki.fi | **Clinical Groups** | **ENT (ear, nose and throath) Group** | |
| Tytti Willberg | Hospital District of Southwest Finland, Turku, Finland | tytti.willberg@tyks.fi | **Clinical Groups** | **ENT (ear, nose and throath) Group** | |
| Elmo Saarentaus | Institute for Molecular Medicine Finland (FIMM), HiLIFE, University of Helsinki, Helsinki, Finland | elmo.saarentaus@helsinki.fi | **Clinical Groups** | **ENT (ear, nose and throath) Group** | |
| Antti Aarnisalo | Hospital District of Helsinki and Uusimaa, Helsinki, Finland | antti.aarnisalo@hus.fi | **Clinical Groups** | **ENT (ear, nose and throath) Group** | |
| Eveliina Salminen | Hospital District of Helsinki and Uusimaa, Helsinki, Finland | eveliina.e.salminen@hus.fi | **Clinical Groups** | **ENT (ear, nose and throath) Group** | |
| Elisa Rahikkala | Northern Ostrobothnia Hospital District, Oulu, Finland | elisa.rahikkala@ppshp.fi | **Clinical Groups** | **ENT (ear, nose and throath) Group** | |
| Johannes Kettunen | Northern Ostrobothnia Hospital District, Oulu, Finland | johannes.kettunen@oulu.fi | **Clinical Groups** | **ENT (ear, nose and throath) Group** | |
| Kristiina Aittomäki | Department of Medical Genetics, Helsinki University Central Hospital, Helsinki, Finland | kristiina.aittomaki@helsinki.fi | **Clinical Groups** | **POI (premature ovarian failure) Group** | |
| Fredrik Åberg | Transplantation and Liver Surgery Clinic, Helsinki University Hospital, Helsinki University, Helsinki, Finland | fredrik.aberg@helsinki.fi | **Clinical Groups** | **LiverScore Group** | |
| Mitja Kurki | Institute for Molecular Medicine Finland (FIMM), HiLIFE, University of Helsinki, Helsinki, Finland; Broad Institute, Cambridge, MA, United States | mkurki@broadinstitute.org | **FinnGen Analysis working group** | **FinnGen Analysis working group** | |
| Samuli Ripatti | Institute for Molecular Medicine Finland (FIMM), HiLIFE, University of Helsinki, Helsinki, Finland | samuli.ripatti@helsinki.fi | **FinnGen Analysis working group** | **FinnGen Analysis working group** | |
| Mark Daly | Institute for Molecular Medicine, Finland (FIMM), HiLIFE, University of Helsinki, Helsinki, Finland; Broad Institute of MIT and Harvard; Massachusetts General Hospital | mark.daly@helsinki.fi | **FinnGen Analysis working group** | **FinnGen Analysis working group** | |
| Juha Karjalainen | Institute for Molecular Medicine Finland (FIMM), HiLIFE, University of Helsinki, Helsinki, Finland | juha.karjalainen@helsinki.fi | **FinnGen Analysis working group** | **FinnGen Analysis working group** | |
| Aki Havulinna | Institute for Molecular Medicine Finland (FIMM), HiLIFE, University of Helsinki, Helsinki, Finland; Finnish Institute for Health and Welfare (THL), Helsinki, Finland | aki.havulinna@helsinki.fi | **FinnGen Analysis working group** | **FinnGen Analysis working group** | |
| Juha Mehtonen | Institute for Molecular Medicine Finland (FIMM), HiLIFE, University of Helsinki, Helsinki, Finland | juha.mehtonen@helsinki.fi | **FinnGen Analysis working group** | **FinnGen Analysis working group** | |
| Priit Palta | Institute for Molecular Medicine Finland (FIMM), HiLIFE, University of Helsinki, Helsinki, Finland | priit.palta@helsinki.fi | **FinnGen Analysis working group** | **FinnGen Analysis working group** | |
| Shabbeer Hassan | Institute for Molecular Medicine Finland (FIMM), HiLIFE, University of Helsinki, Helsinki, Finland | shabbeer.hassan@helsinki.fi | **FinnGen Analysis working group** | **FinnGen Analysis working group** | |
| Pietro Della Briotta Parolo | Institute for Molecular Medicine Finland (FIMM), HiLIFE, University of Helsinki, Helsinki, Finland | pietro.dellabriottaparolo@helsinki.fi | **FinnGen Analysis working group** | **FinnGen Analysis working group** | |
| Wei Zhou | Broad Institute, Cambridge, MA, United States | wzhou@broadinstitute.org | **FinnGen Analysis working group** | **FinnGen Analysis working group** | |
| Mutaamba Maasha | Broad Institute, Cambridge, MA, United States | mmaasha@broadinstitute.org | **FinnGen Analysis working group** | **FinnGen Analysis working group** | |
| Shabbeer Hassan | Institute for Molecular Medicine Finland (FIMM), HiLIFE, University of Helsinki, Helsinki, Finland | shabbeer.hassan@helsinki.fi | **FinnGen Analysis working group** | **FinnGen Analysis working group** | |
| Susanna Lemmelä | Institute for Molecular Medicine Finland (FIMM), HiLIFE, University of Helsinki, Helsinki, Finland | susanna.lemmela@helsinki.fi | **FinnGen Analysis working group** | **FinnGen Analysis working group** | |
| Manuel Rivas | University of Stanford, Stanford, CA, United States | mrivas@stanford.edu | **FinnGen Analysis working group** | **FinnGen Analysis working group** | |
| Aarno Palotie | Institute for Molecular Medicine Finland (FIMM), HiLIFE, University of Helsinki, Helsinki, Finland | aarno.palotie@helsinki.fi | **FinnGen Analysis working group** | **FinnGen Analysis working group** | |
| Aoxing Liu | Institute for Molecular Medicine Finland (FIMM), HiLIFE, University of Helsinki, Helsinki, Finland | aoxing.liu@helsinki.fi | **FinnGen Analysis working group** | **FinnGen Analysis working group** | |
| Arto Lehisto | Institute for Molecular Medicine Finland (FIMM), HiLIFE, University of Helsinki, Helsinki, Finland | arto.lehisto@helsinki.fi | **FinnGen Analysis working group** | **FinnGen Analysis working group** | |
| Andrea Ganna | Institute for Molecular Medicine Finland (FIMM), HiLIFE, University of Helsinki, Helsinki, Finland | aganna@broadinstitute.org | **FinnGen Analysis working group** | **FinnGen Analysis working group** | |
| Vincent Llorens | Institute for Molecular Medicine Finland (FIMM), HiLIFE, University of Helsinki, Helsinki, Finland | vincent.llorens@helsinki.fi | **FinnGen Analysis working group** | **FinnGen Analysis working group** | |
| Hannele Laivuori | Institute for Molecular Medicine Finland (FIMM), HiLIFE, University of Helsinki, Helsinki, Finland | hannele.laivuori@helsinki.fi | **FinnGen Analysis working group** | **FinnGen Analysis working group** | |
| Taru Tukiainen | Institute for Molecular Medicine Finland (FIMM), HiLIFE, University of Helsinki, Helsinki, Finland | taru.tukiainen@helsinki.fi | **FinnGen Analysis working group** | **FinnGen Analysis working group** | |
| Mary Pat Reeve | Institute for Molecular Medicine Finland (FIMM), HiLIFE, University of Helsinki, Helsinki, Finland | mary.reeve@helsinki.fi | **FinnGen Analysis working group** | **FinnGen Analysis working group** | |
| Henrike Heyne | Institute for Molecular Medicine Finland (FIMM), HiLIFE, University of Helsinki, Helsinki, Finland | hheyne@broadinstitute.org | **FinnGen Analysis working group** | **FinnGen Analysis working group** | |
| Nina Mars | Institute for Molecular Medicine Finland (FIMM), HiLIFE, University of Helsinki, Helsinki, Finland | nina.mars@helsinki.fi | **FinnGen Analysis working group** | **FinnGen Analysis working group** | |
| Joel Rämö | Institute for Molecular Medicine Finland (FIMM), HiLIFE, University of Helsinki, Helsinki, Finland | joel.ramo@helsinki.fi | **FinnGen Analysis working group** | **FinnGen Analysis working group** | |
| Elmo Saarentaus | Institute for Molecular Medicine Finland (FIMM), HiLIFE, University of Helsinki, Helsinki, Finland | elmo.saarentaus@helsinki.fi | **FinnGen Analysis working group** | **FinnGen Analysis working group** | |
| Hanna Ollila | Institute for Molecular Medicine Finland (FIMM), HiLIFE, University of Helsinki, Helsinki, Finland | hanna.m.ollila@helsinki.fi | **FinnGen Analysis working group** | **FinnGen Analysis working group** | |
| Rodos Rodosthenous | Institute for Molecular Medicine Finland (FIMM), HiLIFE, University of Helsinki, Helsinki, Finland | rodos.rodosthenous@helsinki.fi | **FinnGen Analysis working group** | **FinnGen Analysis working group** | |
| Satu Strausz | Institute for Molecular Medicine Finland (FIMM), HiLIFE, University of Helsinki, Helsinki, Finland | satu.strausz@helsinki.fi | **FinnGen Analysis working group** | **FinnGen Analysis working group** | |
| Tuula Palotie | University of Helsinki and Hospital District of Helsinki and Uusimaa, Helsinki, Finland | tuula.palotie@helsinki.fi | **FinnGen Analysis working group** | **FinnGen Analysis working group** | |
| Kimmo Palin | University of Helsinki, Helsinki, Finland | kimmo.palin@helsinki.fi | **FinnGen Analysis working group** | **FinnGen Analysis working group** | |
| Javier Garcia-Tabuenca | University of Tampere, Tampere, Finland | javier.graciatabuenca@tuni.fi | **FinnGen Analysis working group** | **FinnGen Analysis working group** | |
| Harri Siirtola | University of Tampere, Tampere, Finland | harri.siirtola@tuni.fi | **FinnGen Analysis working group** | **FinnGen Analysis working group** | |
| Tuomo Kiiskinen | Institute for Molecular Medicine Finland (FIMM), HiLIFE, University of Helsinki, Helsinki, Finland | tuomo.kiiskinen@helsinki.fi | **FinnGen Analysis working group** | **FinnGen Analysis working group** | |
| Jiwoo Lee | Institute for Molecular Medicine Finland (FIMM), HiLIFE, University of Helsinki, Helsinki, Finland; Broad Institute, Cambridge, MA, United States | jiwoo.lee@helsinki.fi | **FinnGen Analysis working group** | **FinnGen Analysis working group** | |
| Kristin Tsuo | Institute for Molecular Medicine Finland (FIMM), HiLIFE, University of Helsinki, Helsinki, Finland; Broad Institute, Cambridge, MA, United States | kristintsuo@fas.harvard.edu | **FinnGen Analysis working group** | **FinnGen Analysis working group** | |
| Amanda Elliott | Institute for Molecular Medicine Finland (FIMM), HiLIFE, University of Helsinki, Helsinki, Finland; Broad Institute, Cambridge, MA, USA and Massachusetts General Hospital, Boston, MA, USA | aelliott@broadinstitute.org | **FinnGen Analysis working group** | **FinnGen Analysis working group** | |
| Kati Kristiansson | THL Biobank / Finnish Institute for Health and Welfare (THL), Helsinki, Finland | kati.kristiansson@thl.fi | **FinnGen Analysis working group** | **FinnGen Analysis working group** | |
| Mikko Arvas | Finnish Red Cross Blood Service / Finnish Hematology Registry and Clinical Biobank, Helsinki, Finland | mikko.arvas@veripalvelu.fi | **FinnGen Analysis working group** | **FinnGen Analysis working group** | |
| Kati Hyvärinen | Finnish Red Cross Blood Service, Helsinki, Finland | kati.hyvarinen@veripalvelu.fi | **FinnGen Analysis working group** | **FinnGen Analysis working group** | |
| Jarmo Ritari | Finnish Red Cross Blood Service, Helsinki, Finland | jarmo.ritari@veripalvelu.fi | **FinnGen Analysis working group** | **FinnGen Analysis working group** | |
| Olli Carpén | Helsinki Biobank / Helsinki University and Hospital District of Helsinki and Uusimaa, Helsinki | olli.carpen@helsinki.fi | **FinnGen Analysis working group** | **FinnGen Analysis working group** | |
| Johannes Kettunen | Northern Finland Biobank Borealis / University of Oulu / Northern Ostrobothnia Hospital District, Oulu, Finland | johannes.kettunen@oulu.fi | **FinnGen Analysis working group** | **FinnGen Analysis working group** | |
| Katri Pylkäs | University of Oulu, Oulu, Finland | katri.pylkas@oulu.fi | **FinnGen Analysis working group** | **FinnGen Analysis working group** | |
| Eeva Sliz | University of Oulu, Oulu, Finland | eeva.sliz@oulu.fi | **FinnGen Analysis working group** | **FinnGen Analysis working group** | |
| Minna Karjalainen | University of Oulu, Oulu, Finland | minna.k.karjalainen@oulu.fi | **FinnGen Analysis working group** | **FinnGen Analysis working group** | |
| Tuomo Mantere | Northern Finland Biobank Borealis / University of Oulu / Northern Ostrobothnia Hospital District, Oulu, Finland | tuomo.mantere@oulu.fi | **FinnGen Analysis working group** | **FinnGen Analysis working group** | |
| Eeva Kangasniemi | Finnish Clinical Biobank Tampere / University of Tampere / Pirkanmaa Hospital District, Tampere, Finland | eeva.kangasniemi@pshp.fi | **FinnGen Analysis working group** | **FinnGen Analysis working group** | |
| Sami Heikkinen | University of Eastern Finland, Kuopio, Finland | sami.heikkinen@uef.fi | **FinnGen Analysis working group** | **FinnGen Analysis working group** | |
| Arto Mannermaa | Biobank of Eastern Finland / University of Eastern Finland / Northern Savo Hospital District, Kuopio, Finland | arto.mannermaa@uef.fi | **FinnGen Analysis working group** | **FinnGen Analysis working group** | |
| Eija Laakkonen | University of Jyväskylä, Jyväskylä, Finland | eija.k.laakkonen@jyu.fi | **FinnGen Analysis working group** | **FinnGen Analysis working group** | |
| Nina Pitkänen | Auria Biobank / University of Turku / Hospital District of Southwest Finland, Turku, Finland | Niina.Pitkanen@tyks.fi | **FinnGen Analysis working group** | **FinnGen Analysis working group** | |
| Samuel Lessard | Translational Sciences, Sanofi R&D, Framingham, MA, USA | samuel.lessard@sanofi.com | **FinnGen Analysis working group** | **FinnGen Analysis working group** | |
| Clément Chatelain | Translational Sciences, Sanofi R&D, Framingham, MA, USA | clement.chatelain@sanofi.com | **FinnGen Analysis working group** | **FinnGen Analysis working group** | |
| Lila Kallio | Auria Biobank / University of Turku / Hospital District of Southwest Finland, Turku, Finland | Lila.Kallio@tyks.fi | **Biobank directors** | **Biobank directors** | |
| Tiina Wahlfors | THL Biobank / Finnish Institute for Health and Welfare (THL), Helsinki, Finland | tiina.wahlfors@thl.fi | **Biobank directors** | **Biobank directors** | |
| Jukka Partanen | Finnish Red Cross Blood Service / Finnish Hematology Registry and Clinical Biobank, Helsinki, Finland | jukka.partanen@veripalvelu.fi | **Biobank directors** | **Biobank directors** | |
| Eero Punkka | Helsinki Biobank / Helsinki University and Hospital District of Helsinki and Uusimaa, Helsinki | eero.punkka@hus.fi | **Biobank directors** | **Biobank directors** | |
| Raisa Serpi | Northern Finland Biobank Borealis / University of Oulu / Northern Ostrobothnia Hospital District, Oulu, Finland | raisa.serpi@ppshp.fi | **Biobank directors** | **Biobank directors** | |
| Sanna Siltanen | Finnish Clinical Biobank Tampere / University of Tampere / Pirkanmaa Hospital District, Tampere, Finland | sanna.siltanen@pshp.fi | **Biobank directors** | **Biobank directors** | |
| Veli-Matti Kosma | Biobank of Eastern Finland / University of Eastern Finland / Northern Savo Hospital District, Kuopio, Finland | veli-matti.kosma@uef.fi | **Biobank directors** | **Biobank directors** | |
| Teijo Kuopio | Central Finland Biobank / University of Jyväskylä / Central Finland Health Care District, Jyväskylä, Finland | teijo.kuopio@ksshp.fi | **Biobank directors** | **Biobank directors** | |
| Anu Jalanko | Institute for Molecular Medicine Finland (FIMM), HiLIFE, University of Helsinki, Helsinki, Finland | anu.jalanko@helsinki.fi | **FinnGen Teams** | **Administration** | |
| Huei-Yi Shen | Institute for Molecular Medicine Finland (FIMM), HiLIFE, University of Helsinki, Helsinki, Finland | huei-yi.shen@helsinki.fi | **FinnGen Teams** | **Administration** | |
| Risto Kajanne | Institute for Molecular Medicine Finland (FIMM), HiLIFE, University of Helsinki, Helsinki, Finland | risto.kajanne@helsinki.fi | **FinnGen Teams** | **Administration** | |
| Mervi Aavikko | Institute for Molecular Medicine Finland (FIMM), HiLIFE, University of Helsinki, Helsinki, Finland | mervi.aavikko@helsinki.fi | **FinnGen Teams** | **Administration** | |
| Helen Cooper | Institute for Molecular Medicine Finland (FIMM), HiLIFE, University of Helsinki, Helsinki, Finland | helen.cooper@helsinki.fi | **FinnGen Teams** | **Administration** | |
| Denise Öller | Institute for Molecular Medicine Finland (FIMM), HiLIFE, University of Helsinki, Helsinki, Finland | denise.oller@helsinki.fi | **FinnGen Teams** | **Administration** | |
| Rasko Leinonen | Institute for Molecular Medicine Finland (FIMM), HiLIFE, University of Helsinki, Helsinki, Finland; European Molecular Biology Laboratory, European Bioinformatics Institute, Cambridge, UK | rasko@ebi.ac.uk | **FinnGen Teams** | **Administration** | |
| Henna Palin | Finnish Clinical Biobank Tampere / University of Tampere / Pirkanmaa Hospital District, Tampere, Finland | henna.palin@pshp.fi | **FinnGen Teams** | **Administration** | |
| Malla-Maria Linna | Helsinki Biobank / Helsinki University and Hospital District of Helsinki and Uusimaa, Helsinki | malla-maria.linna@hus.fi | **FinnGen Teams** | **Administration** | |
| Mitja Kurki | Institute for Molecular Medicine Finland (FIMM), HiLIFE, University of Helsinki, Helsinki, Finland; Broad Institute, Cambridge, MA, United States | mkurki@broadinstitute.org | **FinnGen Teams** | **Analysis** |  |
| Juha Karjalainen | Institute for Molecular Medicine Finland (FIMM), HiLIFE, University of Helsinki, Helsinki, Finland | juha.karjalainen@helsinki.fi | **FinnGen Teams** | **Analysis** |  |
| Pietro Della Briotta Parolo | Institute for Molecular Medicine Finland (FIMM), HiLIFE, University of Helsinki, Helsinki, Finland | pietro.dellabriottaparolo@helsinki.fi | **FinnGen Teams** | **Analysis** |  |
| Arto Lehisto | Institute for Molecular Medicine Finland (FIMM), HiLIFE, University of Helsinki, Helsinki, Finland | arto.lehisto@helsinki.fi | **FinnGen Teams** | **Analysis** |  |
| Juha Mehtonen | Institute for Molecular Medicine Finland (FIMM), HiLIFE, University of Helsinki, Helsinki, Finland | juha.mehtonen@helsinki.fi | **FinnGen Teams** | **Analysis** |  |
| Wei Zhou | Broad Institute, Cambridge, MA, United States | wzhou@broadinstitute.org | **FinnGen Teams** | **Analysis** |  |
| Masahiro Kanai | Broad Institute, Cambridge, MA, United States | mkanai@broadinstitute.org | **FinnGen Teams** | **Analysis** |  |
| Mutaamba Maasha | Broad Institute, Cambridge, MA, United States | mmaasha@broadinstitute.org | **FinnGen Teams** | **Analysis** |  |
| Zhili Zheng | Broad Institute, Cambridge, MA, United States | zhengzhi@broadinstitute.org | **FinnGen Teams** | **Analysis** |  |
| Hannele Laivuori | Institute for Molecular Medicine Finland (FIMM), HiLIFE, University of Helsinki, Helsinki, Finland | hannele.laivuori@helsinki.fi | **FinnGen Teams** | **Clinical Endpoint Development** | |
| Aki Havulinna | Institute for Molecular Medicine Finland (FIMM), HiLIFE, University of Helsinki, Helsinki, Finland; Finnish Institute for Health and Welfare (THL), Helsinki, Finland | aki.havulinna@helsinki.fi | **FinnGen Teams** | **Clinical Endpoint Development** | |
| Susanna Lemmelä | Institute for Molecular Medicine Finland (FIMM), HiLIFE, University of Helsinki, Helsinki, Finland | susanna.lemmela@helsinki.fi | **FinnGen Teams** | **Clinical Endpoint Development** | |
| Tuomo Kiiskinen | Institute for Molecular Medicine Finland (FIMM), HiLIFE, University of Helsinki, Helsinki, Finland | tuomo.kiiskinen@helsinki.fi | **FinnGen Teams** | **Clinical Endpoint Development** | |
| L. Elisa Lahtela | Institute for Molecular Medicine Finland (FIMM), HiLIFE, University of Helsinki, Helsinki, Finland | laura.lahtela@helsinki.fi | **FinnGen Teams** | **Clinical Endpoint Development** | |
| Mari Kaunisto | Institute for Molecular Medicine Finland (FIMM), HiLIFE, University of Helsinki, Helsinki, Finland | mari.kaunisto@helsinki.fi | **FinnGen Teams** | **Communication** | |
| Elina Kilpeläinen | Institute for Molecular Medicine Finland (FIMM), HiLIFE, University of Helsinki, Helsinki, Finland | elina.kilpelainen@helsinki.fi | **FinnGen Teams** | **E-Science** |  |
| Timo P. Sipilä | Institute for Molecular Medicine Finland (FIMM), HiLIFE, University of Helsinki, Helsinki, Finland | timo.p.sipila@helsinki.fi | **FinnGen Teams** | **E-Science** |  |
| Oluwaseun Alexander Dada | Institute for Molecular Medicine Finland (FIMM), HiLIFE, University of Helsinki, Helsinki, Finland | alexander.dada@helsinki.fi | **FinnGen Teams** | **E-Science** |  |
| Awaisa Ghazal | Institute for Molecular Medicine Finland (FIMM), HiLIFE, University of Helsinki, Helsinki, Finland | awaisa.ghazal@helsinki.fi | **FinnGen Teams** | **E-Science** |  |
| Anastasia Kytölä | Institute for Molecular Medicine Finland (FIMM), HiLIFE, University of Helsinki, Helsinki, Finland | anastasia.shcherban@helsinki.fi | **FinnGen Teams** | **E-Science** |  |
| Rigbe Weldatsadik | Institute for Molecular Medicine Finland (FIMM), HiLIFE, University of Helsinki, Helsinki, Finland | rigbe.weldatsadik@helsinki.fi | **FinnGen Teams** | **E-Science** |  |
| Sanni Ruotsalainen | Institute for Molecular Medicine Finland (FIMM), HiLIFE, University of Helsinki, Helsinki, Finland | sanni.ruotsalainen@helsinki.fi | **FinnGen Teams** | **E-Science** |  |
| Kati Donner | Institute for Molecular Medicine Finland (FIMM), HiLIFE, University of Helsinki, Helsinki, Finland | kati.donner@helsinki.fi | **FinnGen Teams** | **Genotyping** |  |
| Timo P. Sipilä | Institute for Molecular Medicine Finland (FIMM), HiLIFE, University of Helsinki, Helsinki, Finland | timo.p.sipila@helsinki.fi | **FinnGen Teams** | **Genotyping** |  |
| Anu Loukola | Helsinki Biobank / Helsinki University and Hospital District of Helsinki and Uusimaa, Helsinki | anu.loukola@hus.fi | **FinnGen Teams** | **Sample Collection Coordination** | |
| Päivi Laiho | THL Biobank / Finnish Institute for Health and Welfare (THL), Helsinki, Finland | paivi.laiho@thl.fi | **FinnGen Teams** | **Sample Logistics** | |
| Tuuli Sistonen | THL Biobank / Finnish Institute for Health and Welfare (THL), Helsinki, Finland | tuuli.sistonen@thl.fi | **FinnGen Teams** | **Sample Logistics** | |
| Essi Kaiharju | THL Biobank / Finnish Institute for Health and Welfare (THL), Helsinki, Finland | essi.kaiharju@thl.fi | **FinnGen Teams** | **Sample Logistics** | |
| Markku Laukkanen | THL Biobank / Finnish Institute for Health and Welfare (THL), Helsinki, Finland | markku.laukkanen@thl.fi | **FinnGen Teams** | **Sample Logistics** | |
| Elina Järvensivu | THL Biobank / Finnish Institute for Health and Welfare (THL), Helsinki, Finland | elina.jarvensivu@thl.fi | **FinnGen Teams** | **Sample Logistics** | |
| Sini Lähteenmäki | THL Biobank / Finnish Institute for Health and Welfare (THL), Helsinki, Finland | sini.lahteenmaki@thl.fi | **FinnGen Teams** | **Sample Logistics** | |
| Lotta Männikkö | THL Biobank / Finnish Institute for Health and Welfare (THL), Helsinki, Finland | lotta.mannikko@thl.fi | **FinnGen Teams** | **Sample Logistics** | |
| Regis Wong | THL Biobank / Finnish Institute for Health and Welfare (THL), Helsinki, Finland | regis.wong@thl.fi | **FinnGen Teams** | **Sample Logistics** | |
| Auli Toivola | THL Biobank / Finnish Institute for Health and Welfare (THL), Helsinki, Finland | auli.toivola@thl.fi | **FinnGen Teams** | **Sample Logistics** | |
| Minna Brunfeldt | THL Biobank / Finnish Institute for Health and Welfare (THL), Helsinki, Finland | minna.brunfeldt@thl.fi | **FinnGen Teams** | **Registry Data Operations** | |
| Hannele Mattsson | THL Biobank / Finnish Institute for Health and Welfare (THL), Helsinki, Finland | hannele.mattsson@thl.fi | **FinnGen Teams** | **Registry Data Operations** | |
| Kati Kristiansson | THL Biobank / Finnish Institute for Health and Welfare (THL), Helsinki, Finland | kati.kristiansson@thl.fi | **FinnGen Teams** | **Registry Data Operations** | |
| Susanna Lemmelä | Institute for Molecular Medicine Finland (FIMM), HiLIFE, University of Helsinki, Helsinki, Finland | susanna.lemmela@helsinki.fi | **FinnGen Teams** | **Registry Data Operations** | |
| Sami Koskelainen | THL Biobank / Finnish Institute for Health and Welfare (THL), Helsinki, Finland | sami.koskelainen@thl.fi | **FinnGen Teams** | **Registry Data Operations** | |
| Tero Hiekkalinna | THL Biobank / Finnish Institute for Health and Welfare (THL), Helsinki, Finland | tero.hiekkalinna@helsinki.fi | **FinnGen Teams** | **Registry Data Operations** | |
| Teemu Paajanen | THL Biobank / Finnish Institute for Health and Welfare (THL), Helsinki, Finland | teemu.paajanen@thl.fi | **FinnGen Teams** | **Registry Data Operations** | |
| Priit Palta | Institute for Molecular Medicine Finland (FIMM), HiLIFE, University of Helsinki, Helsinki, Finland | priit.palta@helsinki.fi | **FinnGen Teams** | **Sequencing Informatics** | |
| Shuang Luo | Institute for Molecular Medicine Finland (FIMM), HiLIFE, University of Helsinki, Helsinki, Finland | shuang.luo@helsinki.fi | **FinnGen Teams** | **Sequencing Informatics** | |
| Tarja Laitinen | Pirkanmaa Hospital District, Tampere, Finland | tarja.laitinen@pshp.fi | **FinnGen Teams** | **Trajectory** |  |
| Mary Pat Reeve | Institute for Molecular Medicine Finland (FIMM), HiLIFE, University of Helsinki, Helsinki, Finland | mary.reeve@helsinki.fi | **FinnGen Teams** | **Trajectory** |  |
| Shanmukha Sampath Padmanabhuni | Institute for Molecular Medicine Finland (FIMM), HiLIFE, University of Helsinki, Helsinki, Finland | sam.padmanabhuni@helsinki.fi | **FinnGen Teams** | **Trajectory** |  |
| Marianna Niemi | University of Tampere, Tampere, Finland | marianna.niemi@tuni.fi | **FinnGen Teams** | **Trajectory** |  |
| Harri Siirtola | University of Tampere, Tampere, Finland | harri.siirtola@tuni.fi | **FinnGen Teams** | **Trajectory** |  |
| Javier Gracia-Tabuenca | University of Tampere, Tampere, Finland | javier.graciatabuenca@tuni.fi | **FinnGen Teams** | **Trajectory** |  |
| Mika Helminen | University of Tampere, Tampere, Finland | mika.helminen@tuni.fi | **FinnGen Teams** | **Trajectory** |  |
| Tiina Luukkaala | University of Tampere, Tampere, Finland | tiina.luukkaala@tuni.fi | **FinnGen Teams** | **Trajectory** |  |
| Iida Vähätalo | University of Tampere, Tampere, Finland | iida.vahatalo@epshp.fi | **FinnGen Teams** | **Trajectory** |  |
| Jyrki Tammerluoto | Institute for Molecular Medicine Finland (FIMM), HiLIFE, University of Helsinki, Helsinki, Finland | jyrki.tammerluoto@helsinki.fi | **FinnGen Teams** | **Data protection officer** | |
| Marco Hautalahti | Finnish Biobank Cooperative - FINBB | marco.hautalahti@finbb.fi | **FinnGen Teams** | **FINBB - Finnish biobank cooperative** | |
| Johanna Mäkelä | Finnish Biobank Cooperative - FINBB | johanna.makela@finbb.fi | **FinnGen Teams** | **FINBB - Finnish biobank cooperative** | |
| Sarah Smith | Finnish Biobank Cooperative - FINBB | sarah.smith@finbb.fi | **FinnGen Teams** | **FINBB - Finnish biobank cooperative** | |
| Tom Southerington | Finnish Biobank Cooperative - FINBB | tom.southerington@finbb.fi | **FinnGen Teams** | **FINBB - Finnish biobank cooperative** | |
| Petri Lehto | Finnish Biobank Cooperative - FINBB | petri.lehto@finbb.fi | **FinnGen Teams** | **FINBB - Finnish biobank cooperative** | |
